# Epstein-Barr Virus Synergizes with BRD7 to Conquer c-Myc-Mediated Viral Latency Maintenance via Chromatin Remodeling

**DOI:** 10.1128/spectrum.01237-22

**Published:** 2023-02-02

**Authors:** Shen Li, Li Yang, Yanling Li, Wenxing Yue, Shuyu Xin, Jing Li, Sijing Long, Wentao Zhang, Pengfei Cao, Jianhong Lu

**Affiliations:** a Hunan Cancer Hospital/the Affiliated Cancer Hospital of Xiangya School of Medicine, Central South University, Changsha, Hunan, China; b Department of Microbiology, School of Basic Medical Science, Central South University, Changsha, Hunan, China; c The Key Laboratory of Carcinogenesis and Cancer Invasion of the Chinese Ministry of Education, NHC Key Laboratory of Carcinogenesis, Cancer Research Institute, Central South University, Changsha, Hunan, China; d Department of Hematology, National Clinical Research Center for Geriatric Disorders, Xiangya Hospital, Central South University, Changsha, Hunan, China; e China-Africa Research Center of Infectious Diseases, Central South University, Changsha, Hunan, China; Oklahoma State University

**Keywords:** Epstein-Barr virus, bromodomain-containing protein 7, EBV-encoded nuclear antigen 1, c-Myc, latency maintenance, chromatin remodeling

## Abstract

Epstein-Barr virus (EBV) switches between latent and lytic phases in hosts, which is important in the development of related diseases. However, the underlying mechanism of controlling the viral biphasic life cycle and how EBV mediates this regulation remain largely unknown. This study identified bromodomain-containing protein 7 (BRD7) as a crucial host protein in EBV latent infection. Based on the chromatin immunoprecipitation (ChIP) sequencing of endogenous BRD7 in Burkitt lymphoma cells, we found that EBV drove BRD7 to regulate cellular and viral genomic loci, including the transcriptional activation of c-Myc, a recently reported regulator of EBV latency. Additionally, EBV-mediated BRD7 signals were enriched around the FUSE (far-upstream sequence element) site in chromosome 8 and the enhancer LOC108348026 in the *lgH* locus, which might activate the c-*Myc* alleles. Mechanically, EBV-encoded nuclear antigen 1 (EBNA1) bound to BRD7 and colocalized at promoter regions of the related genes, thus serving as cofactors for the maintenance of viral latency. Moreover, the disruption of BRD7 decreased the c-Myc expression, induced the BZLF1 expression, and reactivated the lytic cycle. Our findings reveal the unique role of BRD7 to synergize with EBV in maintaining the viral latency state via chromatin remodeling. This study paves the way for understanding the new molecular mechanism of EBV-induced chromatin remodeling and latent-lytic switch, providing novel therapeutic candidate targets for EBV persistent infection.

**IMPORTANCE** When establishing persistent infection in most human hosts, EBV is usually latent. How the viral latency is maintained in cells remains largely unknown. c-Myc was recently reported to act as a controller of the lytic switch, while whether and how EBV regulates it remain to be explored. Here, we identified that BRD7 is involved in controlling EBV latency. We found that EBV-mediated BRD7 was enriched in both the normal promoter regions and the translocation alleles of c-*Myc*, and disruption of BRD7 decreased c-Myc expression to reactivate the lytic cycle. We also demonstrated that EBV-encoded EBNA1 bound to and regulated BRD7. Therefore, we reveal a novel mechanism by which EBV can regulate its infection state by coordinating with host BRD7 to target c-Myc. Our findings will help future therapeutic intervention strategies for EBV infection and pathogenesis.

## INTRODUCTION

Epstein-Barr virus (EBV) persistently infects more than 90% of the human population worldwide (1). EBV infection poses numerous health risks in human hosts, such as infectious mononucleosis, lymphoproliferative disorder, and EBV-related carcinomas (2). After primary infection, EBV establishes long-term persistence within the memory B-cell pool of the immunocompetent host (3). During latent infection of B cells, viral genomes express specific sets of genes and proteins according to the given latency type (4, 5). Therefore, understanding how viral latency is maintained in B cells will help identify new EBV-specific targets and therapeutic strategies for EBV-associated disorders.

EBV is the first confirmed human tumor virus initially observed in cultured Burkitt lymphoma (BL) cells in 1964 (6). BL evolves from a germinal center B cell entering the memory compartment but is stuck proliferating and exhibits a genetic hallmark that the oncogene c-*Myc* translocates from chromosome 8 to the other three immunoglobulin loci (chr.2, -14, and -22) (7, 8), which results in constitutive activation of the c-*myc* gene. The EBV-encoded nuclear antigen 1 (EBNA1) is expressed in EBV-related BL and is linked with latency (9, 10). EBNA1 is the only viral protein found in all EBV-associated malignancies and latency types, and is essential for maintaining the viral episomes during latency in infected host cells (4). Importantly, EBNA1 can bind to regions close to the chromosomal translocation breakpoints in c-*Myc-IgH* enhancer LOC108348026 in Raji cells (11).

As one kind of tumorigenic virus, EBV switches between the latent program and lytic reactivation in response to environmental signaling (12, 13). At both phases, feasible viral and cellular factors involved in EBV-host interactions are required for infection, replication, and pathogenesis (14). EBV controls the expression of c-Myc (15), a viral lytic switch regulator that has been described recently (16). Mechanistically, c-Myc bound specific enhancer boxes in viral sites of lytic replication and coordinated with an interaction network to maintain latency in BL. EBNA1 can also inhibit EBV lytic reactivation in B cells (17).

Importantly, EBV infection links to the precise mechanisms in the epigenetic changes of host chromatin, which leads to the development of new phenotypes in the genomic sequence. Bromodomain-containing proteins (BRDs) are acetyl-lysine readers that recognize the binding sites of histones and play important roles in regulating gene expression (18, 19). BRDs, especially BRD4, mediate transcriptional addiction in cancer cells (20, 21). BRD4 is associated with EBNA1 (22) during EBV infection and regulates the viral life cycle (23). In EBV-positive Burkitt lymphoma, it is unclear what roles of other BRDs in the upstream regulation of dysregulated c-*myc* via virus-mediated induction. In the present study, we analyzed the expression of BRD family members to identify epigenetic factors critical for EBV infection. Within the differentially expressed molecules, we found that BRD7 was upregulated and wondered whether it might be associated with the EBV life cycle like BRD4. We further focused on the direct DNA binding property of BRD7 to identify a category of differentially regulated regions in the host chromatin and viral genome and further substantiated these changes through the coordination with c-Myc and EBNA1 during EBV latent infection in BL cells.

## RESULTS

### BRD7 is upregulated in EBV latently infected cells.

**FIG 1:**
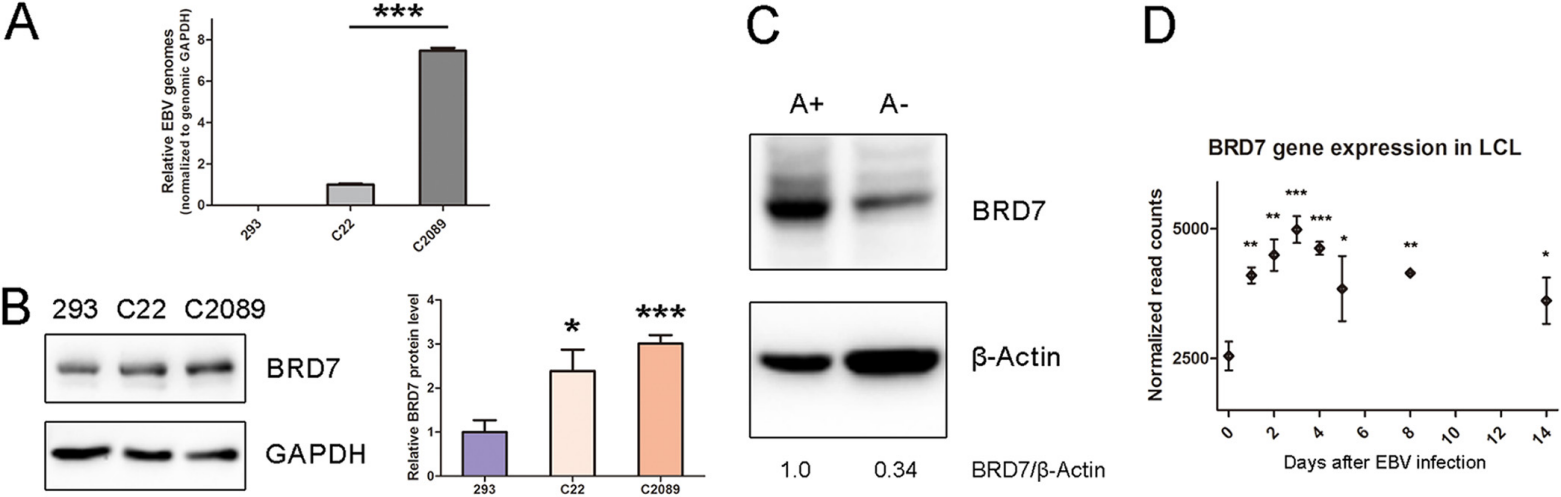
BRD7 is upregulated in latently infected cells with EBV. (A) qPCR analysis of EBV intracellular genome copy number in EBV-negative 293 cells or EBV-positive C22 and C2089 cells. (B) A Western blot assay shows the expression level of BRD7 in 293, C22, and C2089 cells. The expression levels of BRD7 in these three cells were quantified from data from three repeated Western blot assays (right). (C) Expression level of BRD7 in EBV-positive Akata (A^+^) and EBV-negative Akata (A^−^) cells. (D) Based on the database at EBV-b.helmholtz-muenchen.de, the expression of BRD7 in B cells during the time points after EBV infection is shown. For panels A, B, and D, data are expressed as means ± SD from three independent experiments and were analyzed using a two-tailed paired Student's *t* test. *, *P* < 0.05; **, *P* < 0.01; ***, *P* < 0.001.

EBV can cause cellular epigenetic changes in chromatin remodeling via virus-host interactions, including histone acetylation, and lead to infection, replication, and pathogenesis (24). To explore the impact of the bromodomains in EBV latent infection, we analyzed the expression profiles of bromodomain genes based on RNA sequencing that was previously conducted in EBV-negative (EBV^−^ [293]) and EBV latently infected (EBV^+^ [C22 and C2089]) cell lines (25). We identified that bromodomain-containing protein 7 (BRD7), a subunit of the polybromo-associated BAF complexes (PBAF), was upregulated in the EBV^+^ cells (see Fig. S1 in the supplemental material). The EBV copy numbers harbored in these three cell lines for RNA sequencing were different (Fig. 1A). As expected, the BRD7 protein level was confirmed to be increased in the C22 and C2089 cells compared with 293 cells (Fig. 1B). We subsequently investigated the BRD7 expression in the Akata BL cell lines (26). Compared with EBV-negative Akata (A^−^) cells, BRD7 protein abundance was also upregulated in EBV^+^ Akata cells (Fig. 1C). Furthermore, we analyzed the RNA level of BRD7 in latent infection from the database established by Wolfgang Hammerschmidt and his colleagues (27), and the result showed that BRD7 was upregulated in B cells throughout the time points after EBV infection (Fig. 1D). The results implied that BRD7 expression is associated with EBV latent infection.

### EBV mediates BRD7 regulation of the host genome in BL cells.

**FIG 2:**
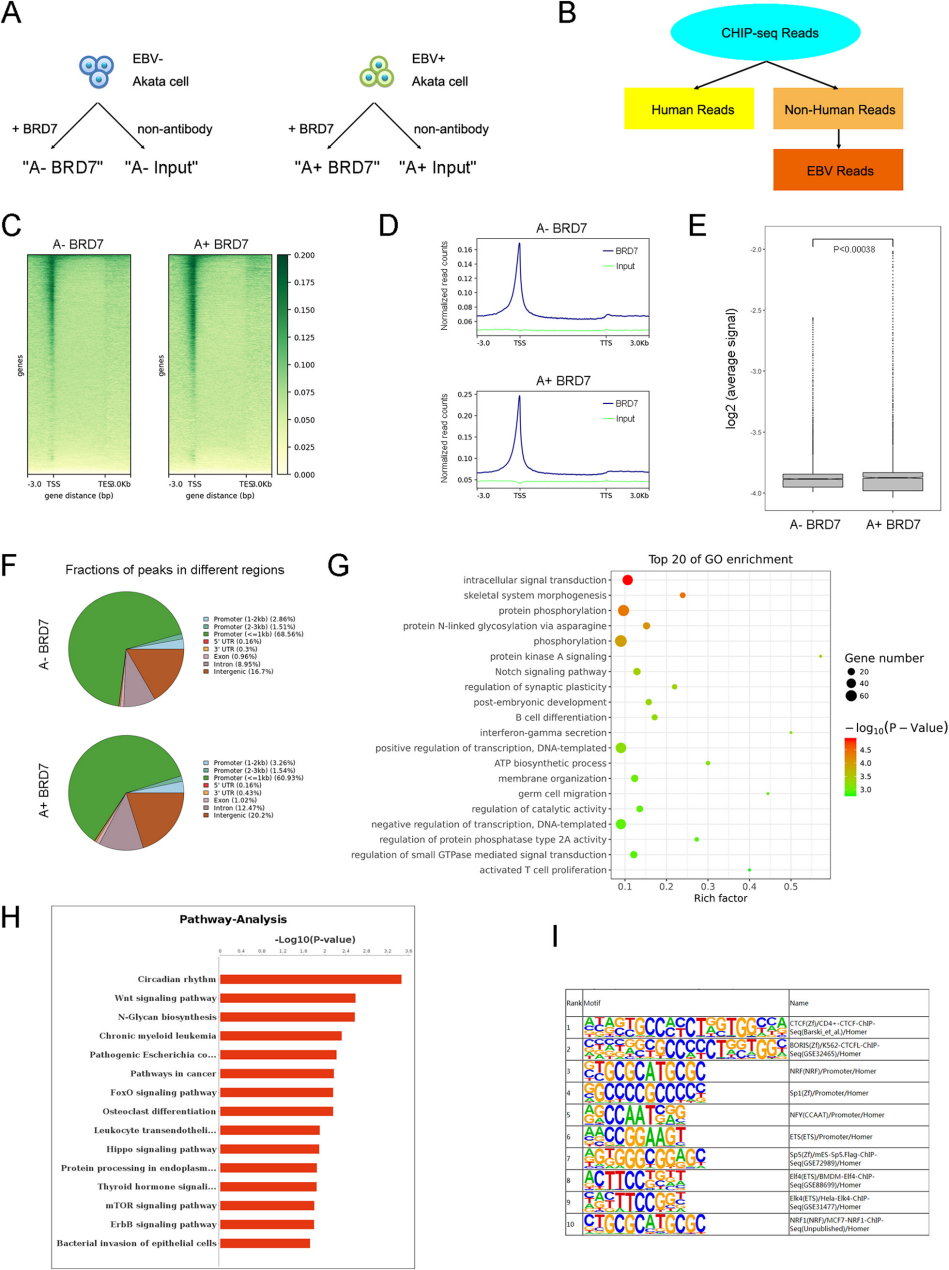
EBV mediates BRD7 regulation of the cellular genome. (A) The schematic depicts the ChIP-seq workflow using EBV^−^ and EBV^+^ Akata cells. (B) Data analysis pipeline for ChIP-seq. All ChIP-seq data were mapped to the human (hg38) or EBV (Akata) genome. (C) Heat map of BRD7 signal around TSSs and the transcriptional end sites (TESs) of all genes in EBV^+^ and EBV^−^ Akata cells. (D) BRD7 enrichment distributions (from bigwig) across genes are presented as an average plot (average read signals across all genes) in EBV^+^ and EBV^−^ Akata cells. (E) A box plot shows the log_2_ average BRD7 signal distribution in regions exhibiting the sites from 3 kb upstream of TSSs to 3 kb downstream of the TESs in EBV^+^ and EBV^−^ Akata cells. (F) Fractions of BRD7 ChIP-seq peaks across different genome regions in EBV^+^ and EBV^−^ Akata cells. (G) GO analysis of BRD7 enrichment for peaks near TSSs of the top 20 genes in EBV^+^ Akata cells compared with EBV^−^ Akata cells. (H) Top 15 pathways of BRD7 enrichment for peaks near the TSSs in EBV^+^ Akata cells compared with EBV^−^ Akata cells. (I) DNA binding motif analysis of the top 10 BRD7-enriched motifs in EBV^+^ Akata cells compared with EBV^−^ Akata cells.

BRD7 is known to bind acetylated lysine residues in histones as a component of a chromatin-remodeling complex (28). However, whether EBV in the modulating activity mediates it remains unknown. To further study the influence of the BRD7 in chromatin remodeling by EBV, a chromatin immunoprecipitation sequencing (ChIP-seq) assay was performed on the EBV^+^ and EBV^−^ Akata cell lines. Both the cell lines are derived from BL with the same host genome, and the EBV^+^ cell line exhibits type I latency (29). ChIP-seq data from four groups of these two cell lines, respectively (Fig. 2A), were mapped in two rounds, including the human genome (hg38) and the viral genome (Akata-EBV) (Fig. 2B). First, we focused on the engagement and response to the host chromatin landscape by BRD7 (Fig. S2A). The majority of BRD7 binding sites were enriched around the transcriptional start sites (TSSs) (Fig. 2C) and overlapped in promoter regions of cellular genes (Fig. 2F). Importantly, for those peaks linked to BRD7 upon EBV latent infection in Akata cells, chromatin accessibility around TSSs was markedly increased (Fig. 2D and E).

Recent studies have shown that EBV latent infection initiates energy consumption, entry into the cell cycle, and first cell divisions (27, 30). Next, the effects of EBV regulation on BL cells’ biological implications and pathways through BRD7 were investigated. Based on the Gene Ontology (GO) analysis and pathways analysis (Fig. 2G and H and Fig. S2C to S2F), the BRD7-enriched and upregulated biological processes in EBV^+^ Akata cells are those functioning in protein processing (Fig. S2F), B-cell differentiation (Fig. 2G), and cell cycle and division (Fig. S2D). These data suggested that EBV mediates BRD7 regulation of a core network of genes that control BL development.

According to the reported DNA binding motifs of EBNA1 related to B-cell immortalization, four families, including CTCF/BORIS, SP1, ETS, and IRF, were involved (31). We further explored the possibility that BRD7 may be associated with these specific motifs in chromatin accessibility. The motifs from EBV-mediated BRD7 upregulation peaks from the ChIP-seq data were analyzed. Three family motifs, CTCF/BORIS, SP1, and ETS, were significantly increased in the EBV^+^ Akata cells (Fig. 2I). We did not observe the IRF motif in the top 50 motifs for the upregulation peaks (data not shown) reported as the motifs correlated with the late stage during EBV latency. Additionally, the p53-related motifs were found within the top 20, indicating that p53, the famous tumor suppressor (32), served as a potential cofactor of BRD7 in the EBV latency.

Some transcription factors can form with large enhancer domains in some genomic sites (also known as superenhancers) to initiate the transcription of those genes that are important for the pluripotent state in embryonic stem cells (33). In addition, some EBVs’ transcription factors drive cellular gene expression through long-range enhancer-promoter looping to activate key oncogenes in B cells (15, 34). Our study found that the BRD7 signals were associated with 1,914 typical enhancers and 26 superenhancers in EBV^+^ Akata cells (Fig. S2G and S2H) versus 374 typical enhancers and 10 superenhancers in EBV^−^ Akata cells (Fig. S2I to S2J). These data indicated that EBV could exert its effect on the transcriptional regulation from cellular enhancer sites through BRD7.

**FIG 3:**
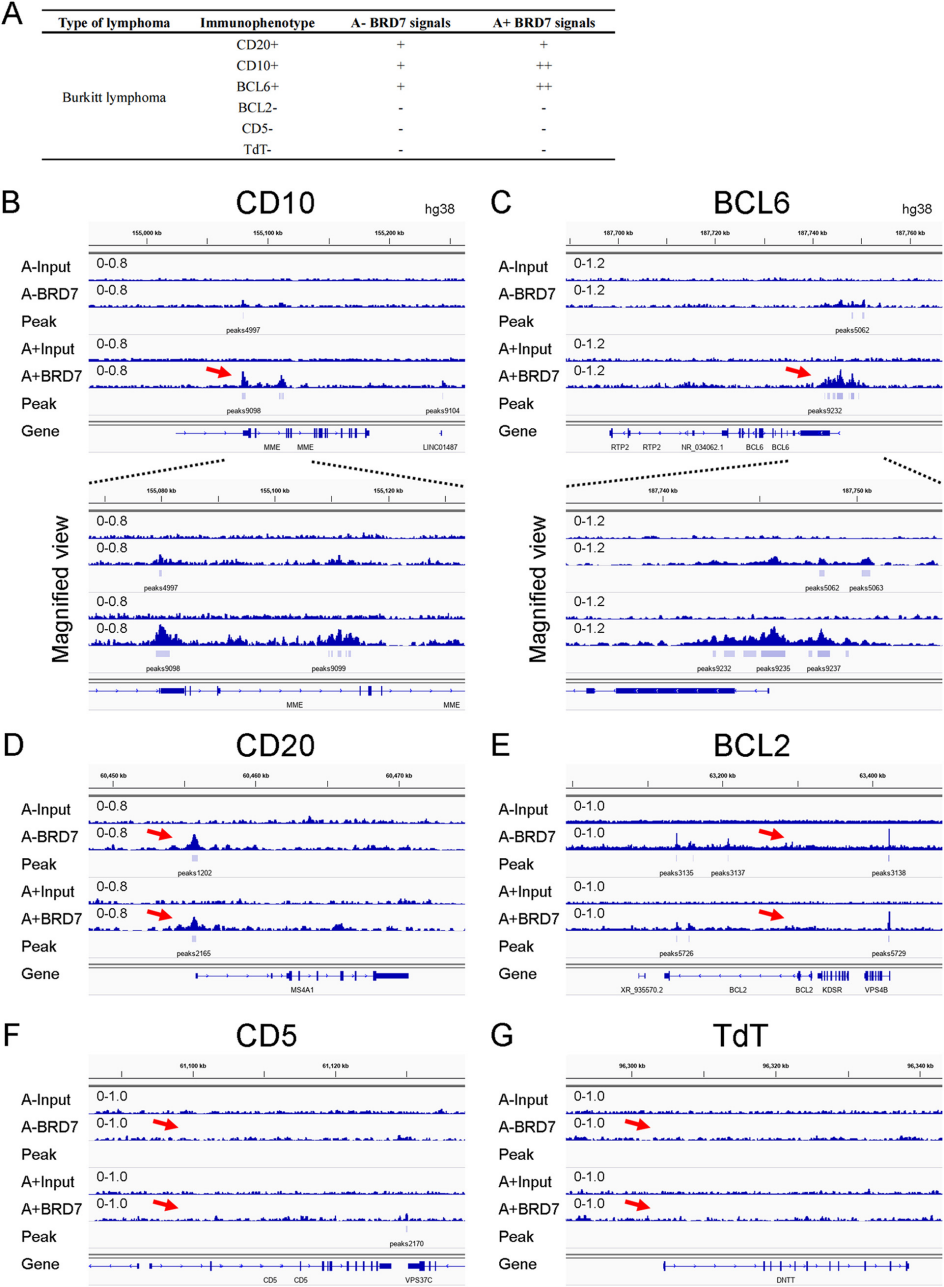
BRD7 signals at genes known to be important for BL. (A) BRD7 peaks in BL genes in Akata cells. Tracks show BRD7 ChIP-seq signals at *CD10* (B), *BCL6* (C), *CD20* (D), *BCL2* (E), *CD5* (F), and *TdT* (G) in Akata cells. Red arrows show the TSSs of the indicated genes.

With the aid of histochemistry, defining features of BL cells are positive for CD20, CD10, and BCL6 but negative for BCL2, CD5, and TdT (35, 36). We initially surveyed the BRD7 ChIP-seq signals at genes important for BL. BRD7 peaks in Akata cells were found at the BL-associated genes *CD20*, *CD10*, and *BCL6* (Fig. 3A). Importantly, significant BRD7 signals were enriched near the TSSs of *CD10* and *BCL6* in EBV^+^ Akata cells. Additionally, the peaks were absent around the TSSs of *BCL2*, *CD5*, and *TdT* (Fig. 3B to G). The results implied that EBV might mediate the transcriptional activity of *CD10* and *BCL6* through BRD7 in BL cells.

### BRD7 is important for EBV latency maintenance in BL cells.

The EBV genome persists in infected host cells as extrachromosomal episomes and is subject to chromatin-mediated regulation (37), raising our question of whether BRD7 may regulate the viral genomes in latent infection. In the above human genome mapping analysis, nonhuman sequences are involved (Fig. S2A). Based on the annotation of viral genomes from EBV^+^ Akata BL cells (GenBank accession no. KC207813.1), 7.56% of total reads from EBV^+^ Akata BRD7 ChIP-seq data were mapped to the viral genome (Fig. S2B). The data implied that binding of the host chromatin-remodeling protein BRD7 to the EBV genome has an established role in maintaining viral latency.

**FIG 4:**
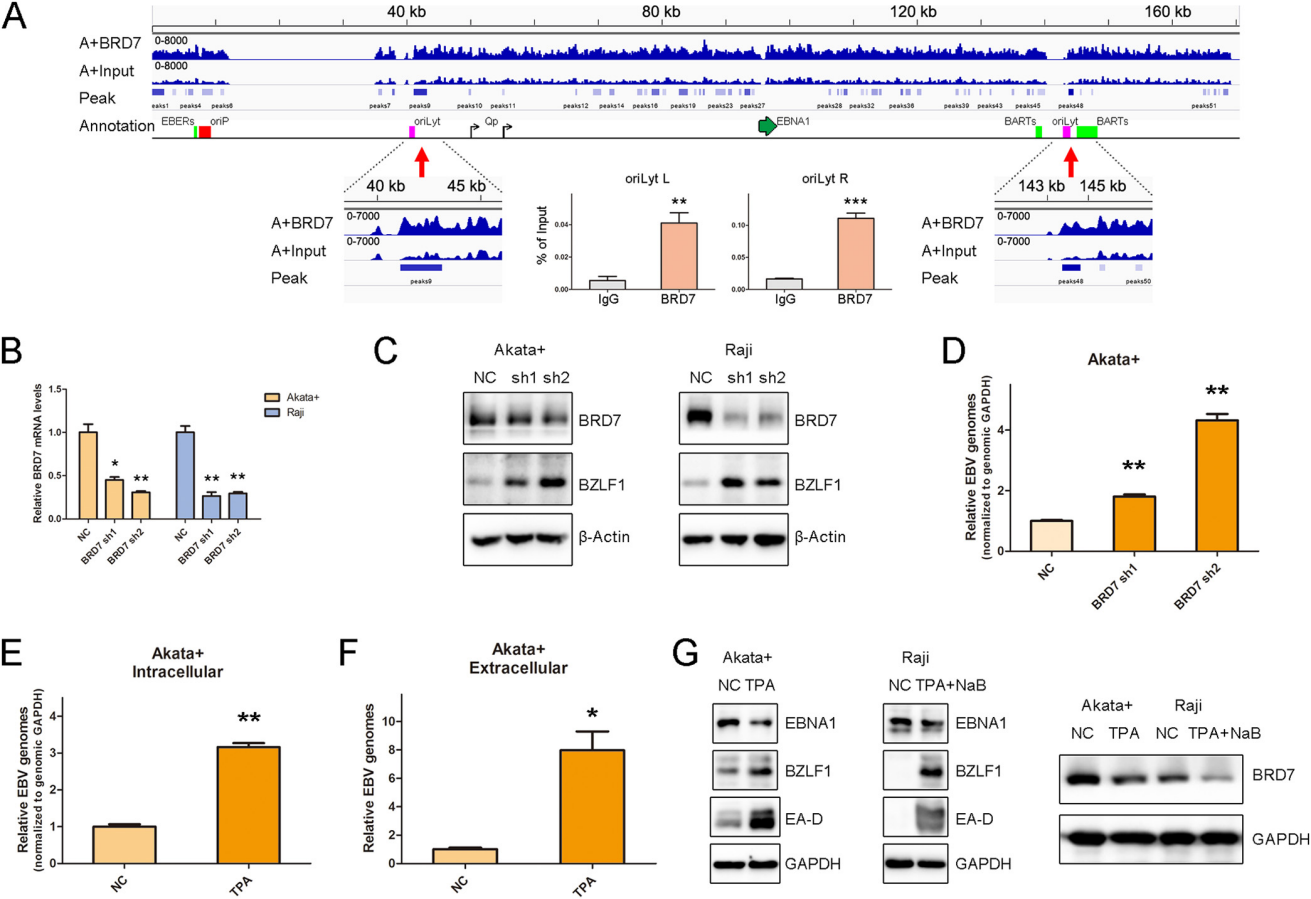
BRD7 binds to replication lytic origins and maintains BL's EBV latency. (A) BRD7 binds specific sites across the viral genome (GenBank accession no. KC207813.1). Red arrows indicate enlarged images for BRD7 binding sites at the oriLyt loci. Shown are the results from ChIP-qPCR analysis of BRD7 occupancy at oriLyt regions. The input and anti-IgG antibodies were used as positive and negative controls, respectively. (B) RT-PCR analysis of *BRD7* mRNA level in EBV^+^ Akata and Raji cells with control or BRD7 stable knockdown (shRNAs). The *GAPDH* (glyceraldehyde-3-phosphate dehydrogenase) gene served as an internal RNA control in RT-qPCR analysis. (C) Western blot shows BRD7 and BZLF1 protein levels in EBV^+^ Akata and Raji cells with control or BRD7 shRNAs. For the quantification of BZLF1, data were used from three independent Western blot experiments. (D) qPCR analysis of EBV intracellular genome copy number from EBV^+^ Akata cells with control or BRD7 shRNAs. (E) qPCR analysis of EBV intracellular genome copy number in EBV^+^ Akata cells treated with the control or TPA for 24 h. (F) qPCR analysis of EBV extracellular genome copy number in EBV^+^ Akata cells treated with the control or TPA for 24 h. (G) A Western blot shows the expression levels of EBNA1, BZLF1, EA-D, and BRD7 in EBV^+^ Akata cells treated with the control or TPA for 24 h. For Raji cells, NaB was added to the TPA treatment. Data are expressed as means ± SD from three independent experiments and were analyzed using a two-tailed paired Student's *t* test. *, *P* < 0.05; **, *P* < 0.01; ***, *P* < 0.001.

To understand the location of BRD7 binding sites on the viral genome preferentially, we identified the viral sequences in the locations of the viral genome. We found that BRD7 could interact with some sites in the viral genome, centered on the origins of EBV lytic replication (oriLyt sites) (Fig. 4A) (38). This raised the question of whether BRD7, highly induced during EBV latent infection, could be a key regulator of the viral lytic program. Therefore, two BL cell lines with stable BRD7 knockdown were generated using lentivirus-mediated short hairpin RNAs (shRNAs), validated by reverse transcription-quantitative PCR (RT-qPCR) assay (Fig. 4B). The result showed that the knockdown of BRD7 in EBV^+^ Akata and Raji cells significantly increased the expression of BZLF1, a master promoter of the viral lytic cycle (Fig. 4C) (14, 39). EBV intracellular genome copy numbers in EBV^+^ Akata cells increased with BRD7 knockdown (Fig. 4D). These data showed that BRD7 might suppress EBV lytic reactivation in BL cells.

The virus is primarily latent in EBV^+^ Burkitt lymphoma cells (13). The virus can be efficiently transitioned into the lytic phase through the indicated regents in many of these cell systems, making them ideal for certain lytic cycle-based investigations. Lytic induction by 12-*O*-tetradecanoylphorbol 13-acetate (TPA) triggered BZLF1 expression (Fig. S3A) and stimulated viral genome replication in latently infected Akata cells (40). In contrast, the TPA treatment alone does not enhance BZLF1 expression in Raji cells (Fig. S3B). In addition, the viral genome in the Raji line lacks some lytic genes and thus is replication incompetent (41). To further substantiate the regulation of BRD7 on the viral life cycle, we treated EBV^+^ Akata cells with TPA (Fig. 4E and F) and Raji cells with TPA and sodium butyrate (NaB) (42) (Fig. S3C and S3D). The result showed that BRD7 protein abundance was significantly diminished in EBV lytic reactivation (Fig. 4G and Fig. S3E). These data suggested that BRD7 is an adverse factor for EBV lytic replication in BL cells.

### EBV mediates BRD7 regulation of c-*Myc* by binding specific regulatory elements.

A recent study reported that a CRISPR-Cas9-based knockout screen in EBV^+^ BL cells had identified c-Myc as a key mediator of EBV latency (16). This report prompted us to investigate whether BRD7 was involved in c-Myc-related regulation activity, specifically DNA binding mechanisms. The transcriptional regulation of the normal c-*Myc* gene is controlled by the far-upstream sequence element (FUSE) (43). Importantly, BL harbors a classic mutation of an allele that translocates c-*MYC* into the vicinity of one of the immunoglobulin gene loci, causing deregulated c-*Myc* enhancement under the control of active Ig *cis*-regulatory elements, including the *IgH* 3′ regulatory region, the *IgH* intronic enhancer (Eμ), and the enhancer LOC108348026 (11, 44, 45).

**FIG 5:**
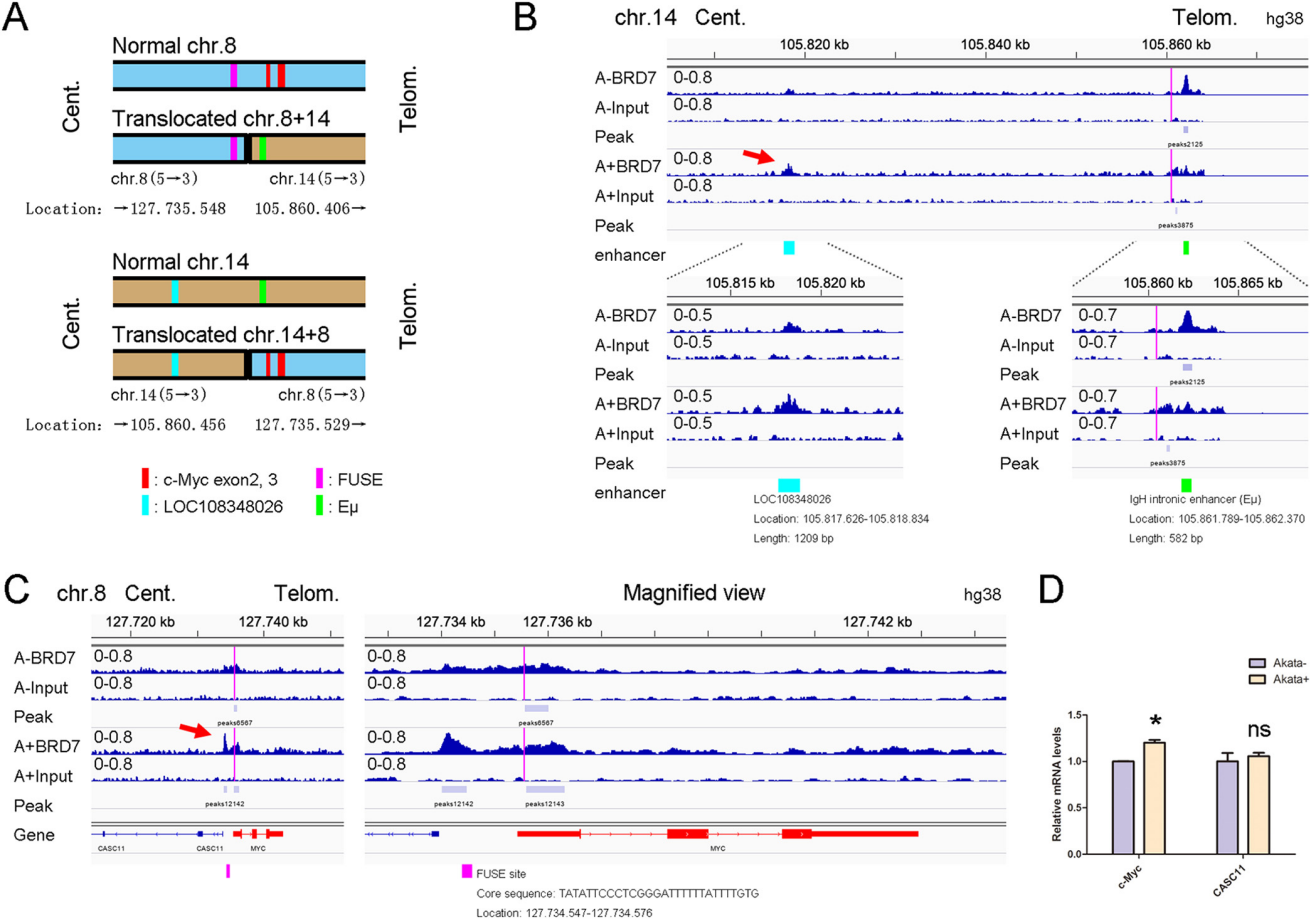
EBV mediates BRD7 regulation of c-*Myc* transcriptional activation. (A) Schematic display of the translocated c-*Myc* allele and location of breakpoint regions in 8q24 and 14q32. The two colors in one chromosome indicate the genomic regions of each translocated chromosomal partner. Red bars indicate c-*Myc* exon2 and exon3. Pink bars show the FUSE site. Blue bars show enhancer LOC108348026. Green bars show the *IgH* intronic enhancer (Eμ). (B) Tracks show BRD7 enriched around the enhancer LOC108348026 (red arrow) in EBV^+^ Akata cells and the enhancer Eμ in EBV^−^ Akata cells. Red lines indicate the exact breakpoint of the translocation on 14q32. Cyan and green boxes show the locations of the indicated enhancer. (C) Tracks show BRD7 peaks in the promoter region 1.5 kb upstream (red arrow) of the c-*Myc* TSS in EBV^+^ Akata cells. Red lines indicate the exact breakpoint of the translocation. (D) RT-qPCR analysis of c-*Myc* and *CASC11* mRNA levels in EBV^−^ and EBV^+^ Akata cells. Data are expressed as means ± SD from three independent experiments and analyzed using a two-tailed paired Student's *t* test. *, *P* < 0.05; ns, not significant.

Based on multiround analyses from ChIP-seq raw data of the Input groups, we found two key reads from ~160 million reads (Fig. S4A) and identified the locations of c-*Myc-IgH* translocation breakpoints on chromosomes 8 and 14 in Akata cells (Fig. S4B), the first description of the exact breakpoint in Akata cells, of which chromosome 8 is located in the first exon of c-*Myc* and chromosome 14 is located between the enhancer Eμ and LOC108348026. The exact location of the breakpoints perhaps indicated that the dysregulation of c-*Myc* is relevant for the enhancer LOC108348026 but not the enhancer Eμ (Fig. 5A). In contrast, our data revealed that EBV-mediated BRD7 could distinctly bind to the region in *IgH* enhancer LOC108348026, defined as a tissue-specific transcribed enhancer in B cells (46) and as an EBNA1 binding site in Raji cells (11). Interestingly, the BRD7 enrichment signal in the enhancer Eμ was significantly decreased in EBV^+^ Akata cells compared with EBV^−^ cells (Fig. 5B).

Additionally, we found that EBV-mediated BRD7 was highly enriched in the region of 1.5 kb upstream of the normal c-*Myc* allele TSS (Fig. 5C), which is also located in the FUSE region (47–49). We further evaluated the expression levels of c-*Myc* and cancer susceptibility candidate 11 (*CASC11*) located in the chromosome 8q24 gene desert (2.1 kb upstream of c-*Myc*). The expression of c-*Myc* was upregulated in EBV^+^ Akata cells, whereas the expression level of *CASC11*, the neighbor gene of c-*Myc*, was of no significant difference (Fig. 5D). These data raised the possibility of an epigenetic mechanism involving c-*Myc* transcriptional activation through BRD7 in EBV latently infected BL cells.

### BRD7 is associated with c-Myc in latent infection.

**FIG 6:**
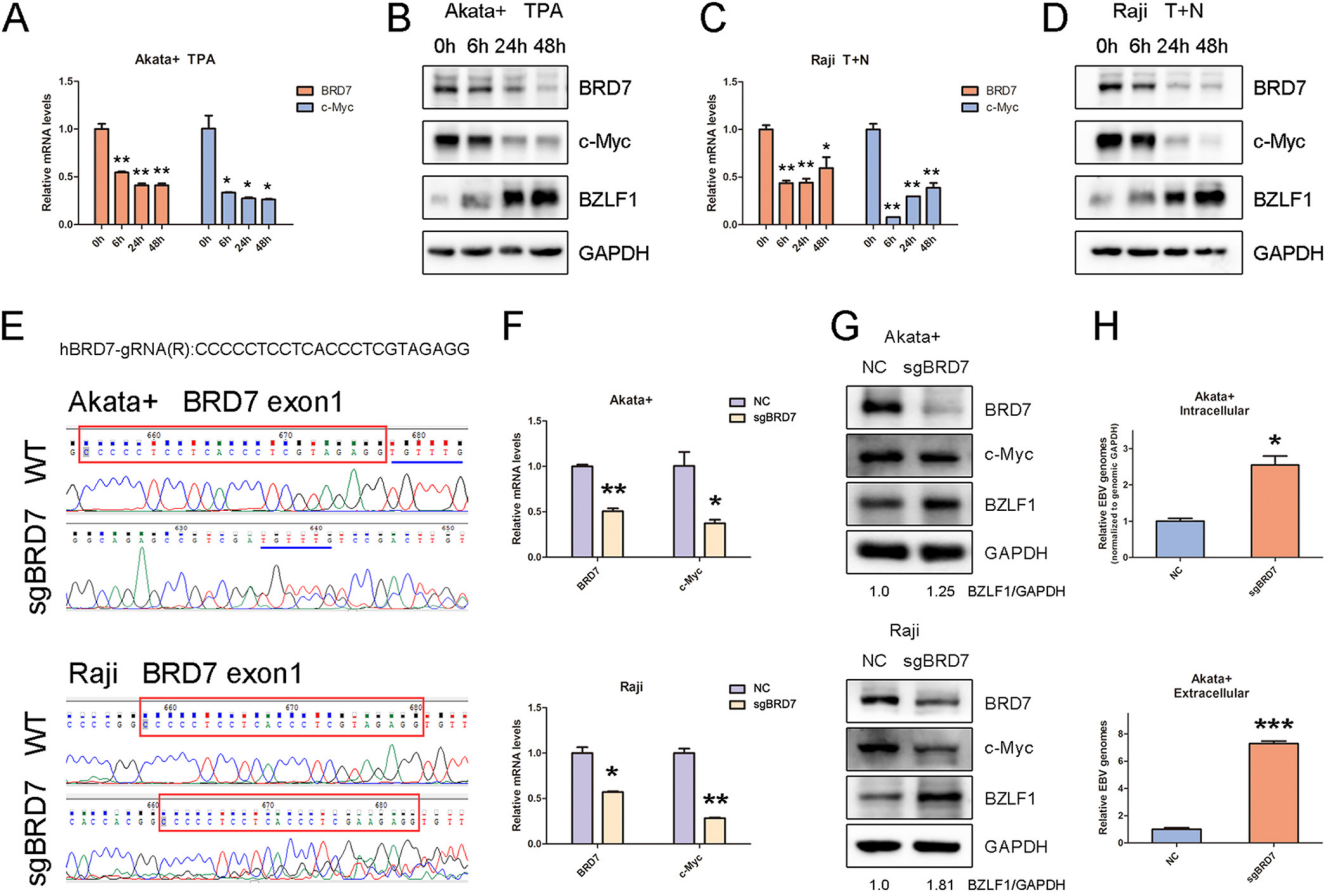
BRD7 is associated with c-Myc during EBV latency. (A) RT-qPCR analysis of *BRD7* and c-*Myc* mRNA levels in EBV^+^ Akata cells with TPA treatment for the indicated number of hours. (B) Western blot of whole-cell lysates (WCL) from EBV^+^ Akata cells with TPA treatment for the indicated number of hours. (C) RT-qPCR analysis of *BRD7* and c-*Myc* mRNA levels in Raji cells treated with TPA plus NaB (T+N) for the indicated times. (D) Western blot of WCL from Raji cells treated with TPA plus NaB for the indicated times. (E) Sanger sequencing data show BRD7-targeted genome editing. Cells overexpressing Cas9 with no guide RNAs were used as negative controls (NC). Red boxes indicate the on-target sequence in the BRD7 exon 1 locus. Blue lines show the indicated location in EBV^+^ Akata cells with control or BRD7 sgRNA knockout. (F) RT-qPCR analysis of *BRD7* and c-*Myc* mRNA levels in EBV^+^ Akata and Raji cells with control or BRD7 knockout. (G) Western blot of whole-cell lysates from EBV^+^ Akata and Raji cells with the control or BRD7 knockout. (H) qPCR analysis of EBV intracellular and extracellular genome copy number from EBV^+^ Akata cells with the control or BRD7 knockout. Data are expressed as means ± SD from three independent experiments and were analyzed using a two-tailed paired Student's *t* test. *, *P* < 0.05; **, *P* < 0.01; ***, *P* < 0.001.

A further test of four time points was used to observe the responses of BRD7 and c-Myc expression to EBV lytic reactivation. The result showed that the transcriptional levels of both BRD7 and c-Myc were significantly decreased in EBV^+^ Akata and Raji cells during EBV lytic induction (Fig. 6A and C), which was confirmed by immunoblotting (Fig. 6B and D).

A previous study indicated that BRD7 does not directly impact the c-*Myc* mRNA level in colorectal cancer cells (50). Therefore, regulating the c-*Myc* transcriptional level accompanied by the change of BRD7 expression might depend on EBV latent infection. BRD7-targeting siRNAs were transfected into EBV-negative 293 and EBV-positive C2089 cells to validate this hypothesis (Fig. S5A and S5B). Interestingly, with the knockdown of BRD7, the RNA and protein levels of c-Myc were decreased only in C2089 cells but not in 293 cells (Fig. S5C and S5D). These results also verified that the BRD7-regulated c-Myc depended on EBV infection.

Single guide RNAs (sgRNAs) were used to knock out BRD7 exon 1 in EBV^+^ Akata and Raji cells through the CRISPR-Cas9 technique to verify this observation further. The CRISPR-mediated BRD7 depletion was validated by Sanger sequencing (Fig. 6E). The result also demonstrated that BRD7 depletion reduced the abundance of c-Myc at both RNA and protein levels in EBV^+^ BL cells (Fig. 6F and Fig. S5E). Simultaneously, BRD7 depletion triggered the protein expression of BZLF1 and the EBV genome replication (Fig. 6G and H). These data suggested that BRD7 is a regulator upstream of c-Myc in maintaining the EBV latent phase.

**FIG 7:**
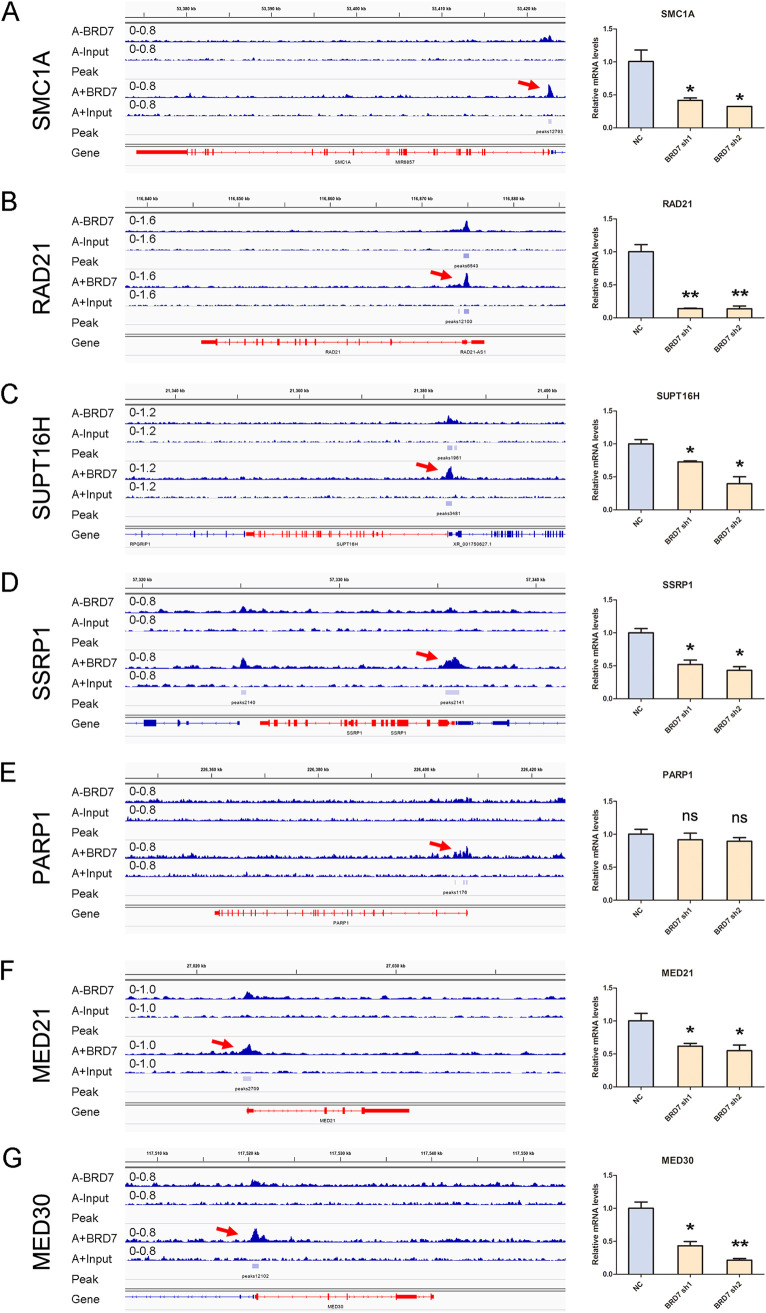
BRD7 binds specific sites of c-Myc-related genes. Tracks show BRD7 occupancy at *SMC1A* (A), *RAD21* (B), *SUPT16H* (C), *SSRP1* (D), *PARP1* (E), *MED21* (F), and *MED30* (G) in EBV^+^ Akata cells. Red arrows indicate the BRD7 binding sites enriched around the TSSs of genes in EBV^+^ Akata cells (left panel). Results of RT-qPCR analysis of indicated mRNA levels in EBV^+^ Akata cells with control or BRD7 stable knockdown are shown in the right panel. Data are expressed as means ± SD from three independent experiments and were analyzed using a two-tailed paired Student's *t* test. *, *P* < 0.05; **, *P* < 0.01; ns, not significant.

The previous work from Gewurz's group has characterized an interaction network (cohesin, FACT, STAGA, and Mediator complex), which supported c-Myc expression to maintain EBV latency in BL cells (16). In the present approach, based on the cooperative functional relationship between BRD7 and c-Myc, we also analyzed key nodes in the network resulting from the BRD7 ChIP-seq data. In comparison with EBV^−^ Akata cells, a series of genes with BRD7 binding sites near their TSSs were identified in EBV^+^ Akata cells, including *SMC1A*, *RAD21*, *SUPT16H*, *SSRP1*, *PARP1*, *MED21*, *MED11*, and *MED30* (cohesin, FACT, and Mediator complex) (Fig. 7). In contrast, BRD7 enrichment does not significantly exist around EP300 and FACT complex (*TADA1*, *TADA2B*, *TADA3*, *TAF5L*, and *SUPT20H*) in EBV^+^ Akata cells (data not shown). Notably, according to the above report with two time points (days 6 and 9) used, subunits of three complexes (PARylation, cohesin, and Mediator) scored more strongly in the early stage, whereas the EP300 and STAGA subunits scored more strongly in the late stage, perhaps reflecting that EBV drives BRD7 to enhance the transcription of EBV latency-related nuclear epigenetic regulators at different time points. These results indicated that BRD7 is a key mediator supporting c-Myc and the specific network of c-Myc-related interactions in maintaining EBV latency in BL.

### EBNA1 regulates and interacts with BRD7 during EBV latency.

**FIG 8:**
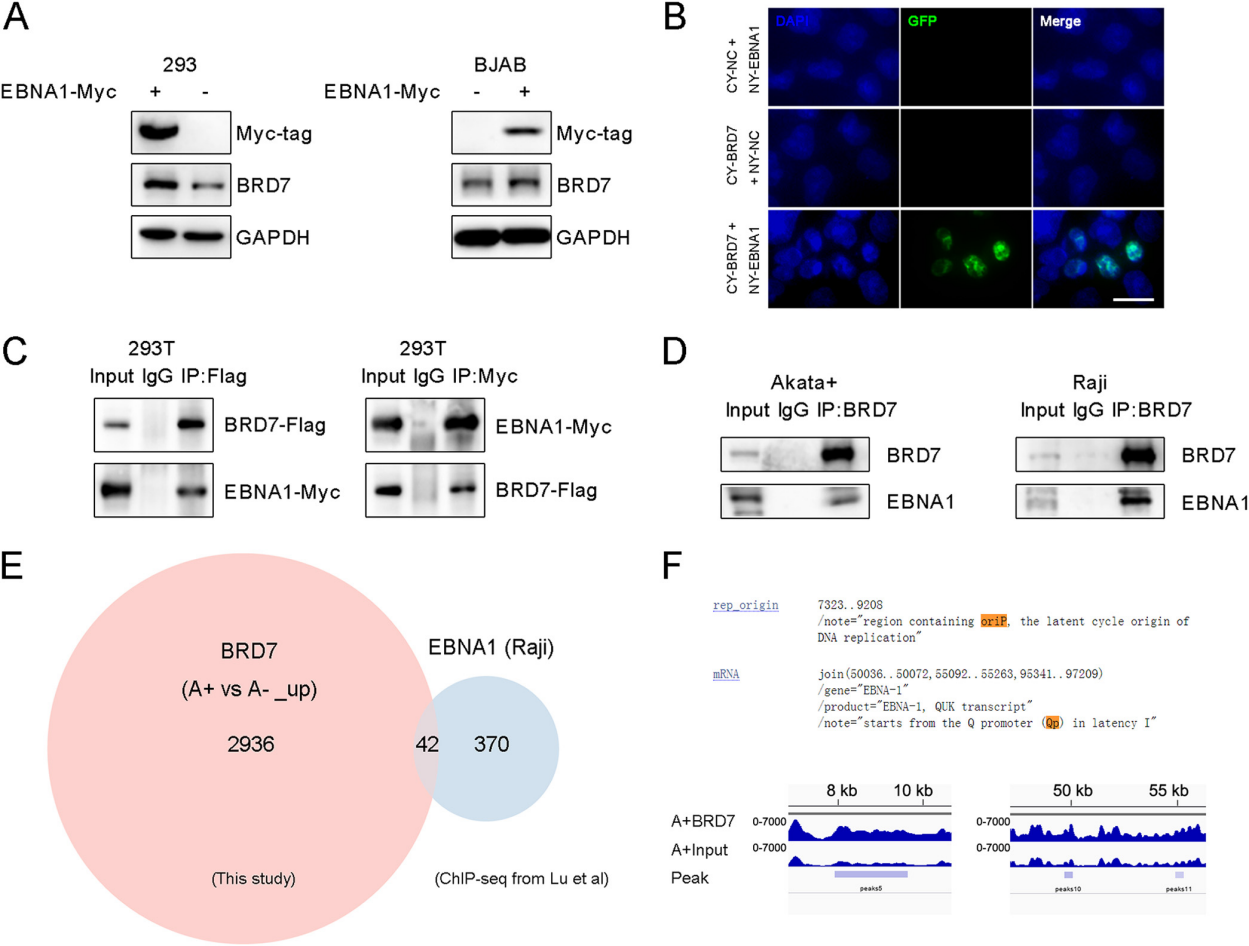
BRD7 is an EBNA1 cofactor in EBV latency regulation. (A) Western blot for Myc-tagged EBNA1 and BRD7 expression in 293 cells transfected with control plasmid or Myc-tagged EBNA1 and BJAB cells transduced with control lentiviral vector or Myc-tagged EBNA1. (B) The BiMC assay shows EBNA1-BRD7 interactions in the nucleus. Scale bar, 20 μm. (C) Binding of exogenous EBNA1 with BRD7 validated by co-IP assay in 293T cells. (D) Binding of endogenous BRD7 and EBNA1 validated by co-IP assay in EBV^+^ Akata and Raji cells. (E) The intersection of cellular genes was obtained from BRD7 ChIP-seq data in EBV^+^ Akata cells compared to those in EBV^−^ Akata cells (>1.2-fold) and EBNA1 ChIP-seq (data are from GSM1905007). Overlapping genes between the groups of EBV upregulated BRD7 and that EBNA1 were analyzed, confirming 42 candidate genes exhibited in these two databases. (F) ChIP-seq for BRD7 across the EBV genome in Akata cells. Red arrows indicate enlarged images at the BRD7-enriched *oriP* and *Qp* sites.

EBNA1 is required for EBV genome replication and maintaining viral episomes during latency (51, 52). It inhibits EBV lytic induction and regulates the host gene expression by binding to the TSS site (11, 17). Because the BRD7 regulation of c-Myc in the viral life cycle depends on EBV infection, we explored the association between EBNA1 and BRD7. The result showed that exogenous expression of EBNA1 promoted the BRD7 protein abundance (Fig. 8A).

A bimolecular complementation (BiMC) assay was performed to assess the binding of EBNA1 and BRD7 in the nucleus of 293T cells (Fig. 8B). Next, a coimmunoprecipitation (co-IP) assay was carried out using tagged or endogenous EBNA1. The results verified the binding of EBNA1 and BRD7 in BL cells (Fig. 8C and D).

EBNA1 is also a DNA-binding protein that can regulate various host cellular genes during EBV latency (53). Lieberman’s group has identified the EBNA1 binding sites near the TSSs of 10 genes in BL cells, including *HDAC3*, *MAP3K1*, *SIVA1*, *MYO1C*, *PBX2*, *NIN*, *WASF2*, *MDK*, *CDC7*, and *TFEB* (11), which prompted us to investigate whether BRD7 also regulates these EBNA1-related genes. BRD7 binds with high affinity and specificity to 7 of these genes in EBV^+^ Akata cells, including *MAP3K1*, *SIVA1*, *PBX2*, *NIN*, *WASF2*, *CDC7*, and *TFEB* (Fig. S6).

We next performed Venn analysis to show the correlation between genes associated with EBV-upregulated BRD7 and those associated with EBNA1. A total of 2,978 BRD7-binding genes were significantly higher (>1.2-fold) in EBV^+^ Akata cells than in EBV^−^ Akata cells. Based on a database of ChIP-seq data from Raji cells (11), we also identified 412 genes associated with EBNA1. The overlapping analysis was then performed and confirmed that ~10% (42) of EBNA1-associated genes were linked to EBV-related BRD7 peaks (Fig. 8E). These data supported that BRD7 colocalized with EBNA1 binding sites and indicated that BRD7 served as an EBNA1 cofactor in the transcriptional activation of cellular genes.

EBNA1 is a critical latent viral factor in *trans* and can bind to *oriP* and *Qp* promoter regions with high affinity (11, 52). The enrichment of BRD7 at *oriP* and *Qp* was analyzed and showed that BRD7 binds to these two regions in the EBV genome (Fig. 8F), suggesting that BRD7 at these sites is important for the maintenance of viral episomal genomes during latent infection. In addition, the downregulation of BRD7 in EBV^+^ Akata cells decreased the transcriptional level of EBNA1, reflecting that BRD7 is a presumable regulator of EBNA1 through binding and activating the *Qp* promoter.

## DISCUSSION

**FIG 9:**
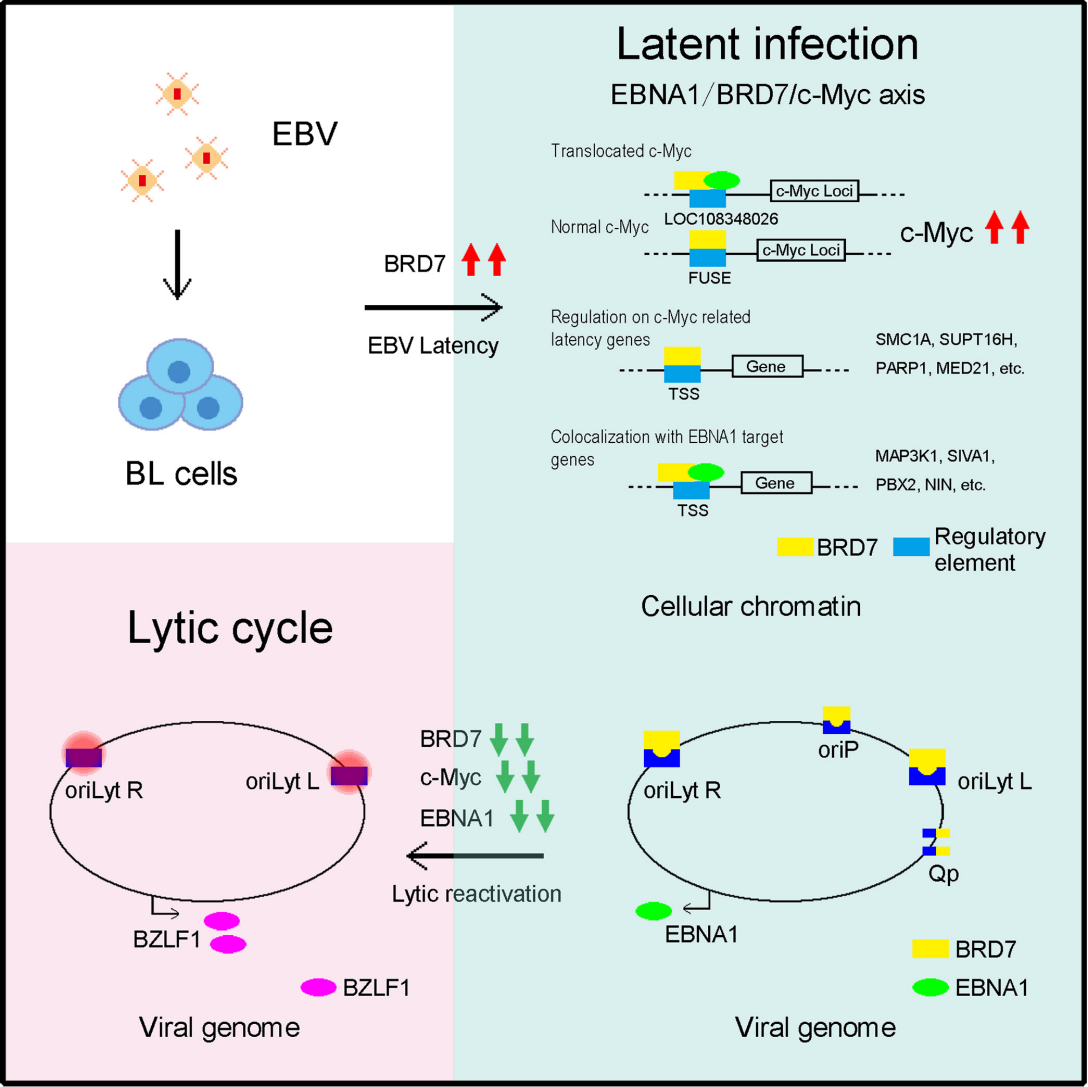
Schematic graphical model. Host BRD7 is upregulated by EBV latent infection. EBV-induced BRD7 alters cellular and viral genome and enhances viral latency by binding with EBNA1 to regulate c-Myc.

With most people infected with EBV globally, regulation of the switch between latency and lytic replication cycles of this virus is critical for understanding EBV-associated diseases (54). The present study finds that BRD7 is a novel host factor to synergize with EBV in regulating cellular and viral genomes, successfully conquering EBV latent-lytic switch via targeting c-*Myc* in BL cells (Fig. 9).

BRD7 is a subunit of PBAF in the mSWI/SNF complex family (55) and has served as a tumor suppressor protein during tumorigenesis in recent years (56). BRD7 is downregulated in nasopharyngeal carcinoma (NPC) (57). Previous studies have shown that c-Myc acts as an upstream negative regulator of BRD7 and inhibits BRD7 expression levels in NPC cells (58, 59). In BL cells, one of the c-*Myc* alleles was translocated into the *lgH* locus, indicating that c-*Myc* alleles are controlled by several elements from two chromosome regions (35, 60). A previous study found c-*Myc* translocation in Akata cells based on their PCR product (61). However, the study should have reported the information on the exact breakpoint. According to our ChIP-seq data, the exact breakpoint information of c-*Myc-IgH* translocation in Akata cells was identified for the first time. In our study, we observed EBV-mediated BRD7 enrichment around the region of the enhancer LOC108348026 of the *IgH* gene locus in chromosome 14 (Fig. 5B). Interestingly, a similar site was also identified as an EBNA1 binding location in Raji cells (11). According to our results in Akata-EBV and those in Raji cells, we summarize this translocation in Fig. S7A. The EBV-induced BRD7 peaks were also highly enriched around the FUSE site, suggesting regulating the normal c-*Myc* transcription (Fig. 5C). In addition, EBV-mediated BRD7 enrichments were dramatically reduced in the enhancer of Eμ, which is located near the breakpoint but not physically interrelated with the c-*Myc* gene (Fig. S7B and S7C). These results imply that an epigenetic mechanism is probably involved in the c-*Myc* transcriptional activation by BRD7 in EBV latently infected BL cells.

The chromatin-mediated epigenetic regulation in the cellular and viral genome is changed during EBV latent-lytic switch (13). Z. Lin and colleagues used the method of lytic induction to amplify viral genomic DNA in EBV^+^ Akata cells and obtained 3.41% of the viral genome versus cellular genome (62). In our ChIP-seq data, the ratio of the viral genome to total reads without lytic induction in EBV^+^ Akata cells was 1.29%, and the ratio in the BRD7 group reached 7.56% (Fig. S2A and S2B). The data indicate that EBV has established roles by colocalizing with BRD7 at the viral genome for its advantage during latent infection.

Notably, our result highlighted an enriched region for BRD7 around the oriLyt sites across the EBV genome (Fig. 4A). When both *oriLyt* sites are present, a similar mechanism also enables c-Myc to occupy these sites and control the maintenance of EBV latency (16). In contrast, another study has demonstrated that BRD4 also can bind the EBV genome at oriLyt sites, whereas BRD4 acts as a promoter of the EBV lytic cycle (23), suggesting that these BRD family members are key regulators of the EBV life cycle by serving as either activator or inhibitor at different viral infection stages.

EBNA1 has been documented to inhibit EBV lytic reactivation (16), and the depletion of EBNA1 leads to increased expression of viral lytic genes, including *BRLF1* and *BZLF1* (17). EBNA1 also mediates long-distance interactions near the point of c-*Myc-IgH* translocation in BL (11). In our study, BRD7 enriched promoter regions of multiple cellular genes in EBV^+^ BL cells, especially for genes related to c-Myc or targeted by EBNA1. BRD7 and EBNA1 colocalize in the EBV genome at both latency regions, *oriP* and *Qp*. Therefore, our results raise a crucial mechanism that BRD7 plays a role in maintaining EBV latency in BL through c-Myc and EBNA1.

This study reveals that BRD7 is essential for maintaining EBV latency in BL cells, elucidating a cooperative role of the EBNA1/BRD7/c-Myc axis in the regulation (Fig. 9). The findings highlight that EBV can control the biphasic life cycle by coordinating with host factors such as BRD7, ultimately providing insights into understanding the viral infection, pathogenesis, and BRD7-based therapeutics for EBV-associated BL.

## MATERIALS AND METHODS

### Cell culture.

The EBV genome (bacterial artificial chromosome [BAC]-based B95-8) (63)-harboring C22 and C2089 cell lines were derived from 293 cells that were previously established in our lab (64). The EBV genome in C22 has an NPC-derived LMP1 gene (GenBank accession no. EF419187) instead of that from B95-8. C22, C2089, EBV^−^ 293, and 293T cells were cultured in Dulbecco's modified Eagle's medium (DMEM) (Gibco), supplemented with 10% fetal bovine serum (Gibco). BJAB, EBV^−^ Akata, EBV^+^ Akata, and Raji cells were cultured in Roswell Park Memorial Institute 1640 medium (RPMI 1640) (Gibco) supplemented with 10% fetal bovine serum. All cell lines were maintained in a humidified atmosphere containing 5% CO_2_ at 37°C.

### Quantification of EBV genome copy number.

To quantify intracellular EBV genome copy number, total intracellular DNA was isolated using the Universal genomic DNA kit (CWbiotech) following the manufacturer's instructions and was primarily quantified with NanoDrop (ThermoFisher Scientific). To quantify extracellular EBV genome copy number, extracellular DNA was isolated from the culture supernatant using the CWhipro circulating nucleic acid kit (CWbiotech). Most notably, the volume of all samples from the culture supernatant remains the same. All qPCR analysis of EBV genome copy numbers was based on two targets: one pair of primers (gDNA-1 or gDNA-2) for the cellular genomic target and the other pair of primers (EBV-W) for the viral genomic target. We recommended using the primers of gDNA-1 for the amplifications as an internal control to rule out cellular targets that contain single nucleotide polymorphisms (SNPs). The primer sequences are listed in Table S1.

### RNA isolation and RT-qPCR analysis.

RT-qPCR assays were performed as described previously (65). Briefly, total RNA was prepared using the TRIzol reagent (CWbiotech, https://www.cwbio.com/goods/index/id/10210) following the manufacturer's instructions. cDNA was generated by reverse transcription of total RNA (1 μg) using RevertAid first-strand cDNA synthesis kit (Thermo Fisher Scientific). RT-qPCR analysis was performed using ChamQ Universal SYBR qPCR master mix (Vazyme Biotech) on a CFX96 Touch real-time PCR detection system (Bio-Rad). The glyceraldehyde-3-phosphate dehydrogenase (*GAPDH*) gene was used for internal normalization, and samples without reverse transcription were used as negative controls. Some primers are designed by PrimerBank (66). The primers used are listed in Table S1.

### Western blot assay.

Briefly, whole-cell lysates were isolated using radioimmunoprecipitation assay (RIPA) lysis buffer plus phenylmethylsulfonyl fluoride (PMSF) (1 mM), and the protein concentration was determined with the bicinchoninic acid (BCA) protein assay kit (Beyotime). Whole-cell lysates were separated by SDS-PAGE, transferred onto the polyvinylidene difluoride (PVDF) membranes (Millipore), blocked with 5% milk in TBST buffer (Tris-buffered saline plus Tween 20), and then incubated with relevant primary antibodies at 4°C overnight, followed by secondary antibody incubation for 1 h at room temperature. The EBNA1 antibody was a product of Santa Cruz Biotech. (sc-81581), all other primary antibodies used in this study are listed in Table S2.

### ChIP-seq.

The ChIP assay was prepared using a ChIP assay kit (Beyotime; P2078) following the manufacturer's instructions. For the BRD7 ChIP assay, the chromatin immunoprecipitations were performed with ChIP formulated BRD7 (D9K2T) rabbit monoclonal antibody (Mab) (CST; no.14910). DNA was purified by DNA clean-up kit (CWbiotech). ChIP libraries were prepared using the NEBNext UltraTMII DNA Library preparation kit (no. E7645S) complemented with NEXTflex DNA barcodes from Bioo Scientific. Ten nanograms of DNA was used as starting material for the input and immunoprecipitation group samples. Libraries were amplified on the thermocycler. Postamplification libraries were size selected at 250 to 450 bp in length using AMPure XP Beads (Beckman Coulter; no. A63881) from Beckman Coulter. ChIP Libraries were validated using Qubit kit and then run on the Illumina Novoseq6000 sequencer.

### ChIP-seq data analyses.

**(i) Mapping of paired-end reads.** Before mapping, all raw sequencing reads (FASTQ files) were processed by FastQC (https://www.bioinformatics.babraham.ac.uk/projects/fastqc/) and confirmed with no significant quality issues. Sequenced reads were trimmed for adaptor sequence, masked for low-complexity or low-quality sequence by Trimmomatic, then mapped to reference genome sequences, including the human genome (GRCh38/hg38) and the viral genome (Akata-EBV, GenBank accession no. KC207813.1), using the bwa program.

**(ii) Peak calling.** The bam file generated by the unique mapped reads as an input file, using MACS2 software (https://hbctraining.github.io/Intro-to-ChIPseq/lessons/05_peak_calling_macs.html) for callpeak with cutoff *Q* value of <0.05. The lists of peak calling data (A- BRD7vsInput and A+ BRD7vsInput, “.narrowPeak” file) have been deposited in the NIH GEO omnibus. ChIP-seq signal tracks were visualized through Integrative Genomics Viewer.

To compare the BRD7 signal in EBV^+^ Akata cells with that in EBV^−^ Akata cells, we analyzed differential accessible peaks through 3 steps. First, each sample's peak files were merged using the bedtools software. Second, the counts of the reads over the bed were determined for each sample using bedtools multicov. Finally, differential accessible peaks were assessed using DEGseq. Regions were designated differentially accessible with an absolute value of the log_2_ fold change of >1.0 and a *P* value of <0.05.

**(iii) Heat map analysis.** Read distributions (from bigwig) across genes are presented as a heat map. Genes are represented as lines and sorted in descending order based on signal intensity. The deep tools tool was used for this analysis.

**(iv) Average plot analysis.** Read distributions (from bigwig) across genes are presented as an average plot (average read signals across all genes). The deep tools tool was used for this analysis.

**(v) Distribution analysis.** Peaks were annotated by the function of the annotated peak of ChIPseeker (67). The annotations and plot distribution were obtained using the plotAnnoPie function of ChIPseeker.

**(vi) GO analysis.** Gene Ontology (GO) analysis was performed to elucidate the biological implications of unique genes in the significant or representative profiles of the gene in the experiment (68). We downloaded the GO annotations from the NCBI (http://www.ncbi.nlm.nih.gov/), UniProt (http://www.uniprot.org/), and Gene Ontology (http://www.geneontology.org/) databases. Fisher's exact test was applied to identify the significant GO categories, and the false-discovery rate (FDR) was used to correct the *P* values.

**(vii) Pathway analysis.** According to the KEGG database, pathway analysis was used to determine the genes’ significant pathways. We turned to Fisher's exact test to select the significant pathway, and the significance threshold was defined by *P* value and FDR (69).

**(viii) Motif analysis.** The differential accessible peaks were used for Motif analysis in EBV^+^ Akata cells compared with EBV^−^ Akata cells. The HOMER's findMotifsGenome.pl tool was used for Motif analysis. The input file is the peak file and the genome fasta file. The DNA sequence is extracted according to the peak file, and the sequence is compared with those in the Motif database to obtain the Motif.

**(ix) Enhancer analysis.** Typical enhancers and superenhancers were defined and analyses carried out as described previously (33). We used ROSE software for the J-plot: the *x* axis shows BRD7 ChIP-seq signals' rank order, and the *y* axis shows normalized BRD7 signals. A line of BRD7-related enhancer signals was drawn from the first one with the lowest signal to the last one with the highest signal to determine a diagonal slope in Akata cells.

We used Deeptools for the profile plot. BRD7 ChIP-seq occupancy at typical enhancers and superenhancers was created by mapping read density to the enhancer regions and their corresponding flanking regions. To visualize the length disparity between typical and superenhancer regions, the enhancer region (between its actual start and end) was scaled relative to its median length.

**(x) EBNA1 ChIP-seq data analysis.** EBNA1-associated genes were obtained from the ChIP-seq database (GSM1905007) (2). These genes were identified from peaks as described above. Briefly, 451 peaks were obtained from the raw reads by mapping to the human genome hg38, peak calling, and the differential accessible peak analysis (EBNA1 versus input). A total of 412 EBNA1-associated genes were then annotated from 451 peaks according to ChIPseeker. To compare the EBNA1-related genes with EBV-upregulated BRD7-related genes, we further performed Venn analysis to determine the overlapping ones.

**(xi) Breakpoint analysis.** We used Manta v.1.6.0 (70) to call c-*Myc-IgH* translocation in Akata cells. To create Manta SV calling workflows, we used the following commands: $MANTA_INSTALL_PATH/bin/configManta.py –bam $BAM –referenceFasta $REFERENCE –runDir $OUTPUT. After running the Manta configuration script, the following commands were run: ./runWorkflow.py.

### Lentivirus production and transduction.

The relative DNA fragments (sh-BRD7-1, sh-BRD7-2, and sh-NC) were subcloned into a lentiviral vector, LV10N (U6/mCherry&Puro) (GenePharma, Shanghai, China), to establish BRD7-silenced stable EBV^+^ Akata and Raji cells. Then, the construct containing sh-BRD7 or sh-NC was cotransfected with packaging plasmids into 293T cells. At 48 h posttransfection, the supernatant from lentivirus-producing cells was filtered through 0.45-μm-pore filters and added to six-well plates seeded with EBV^+^ Akata and Raji cells. The infected cells were isolated using puromycin and flow cytometry. The sequences of sh-BRD7 are listed in Table S1.

The cell line stably overexpressing EBNA1 was constructed through the infection of a lentiviral expression vector (LV5, EF-1aF/GFP&Puro) carrying myc-tagged EBNA1 in BJAB cells. The packaged lentivirus was purchased from GenePharma (Shanghai, China). The rest of the procedure is the same as that used for the generation of shBRD7 BL cells described above.

### Lytic reactivation.

For lytic induction in EBV-positive BL cells, Akata cells were treated with 12-*O*-tetradecanoylphorbol 13-acetate (TPA) (200 nM) for 24 h, and Raji cells were treated with TPA (200 nM) and sodium butyrate (NaB) (3 mM) for 24 h. The lytic reactivation was validated by EBV genome copy number quantification and immunoblotting of Zta (the EBV primary latent-lytic switch protein and the product of the *BZLF1* gene, also called Z, ZEBRA, and EB1).

### RNA interference.

Two small interfering RNAs (siRNAs) for BRD7 were designed and synthesized (GenePharma, Shanghai, China). A scrambled sequence served as a negative control (si-NC). The 293 and C2089 cells were transiently transfected with 100 pmol of siRNAs using Lipofectamine 3000 (Invitrogen). At 48 h posttransfection, the cells were harvested or treated for further experiments. The sequences of synthesized siBRD7 are listed in Table S1.

### sgRNA knockout by CRISPR-Cas9.

The sequences of sgBRD7 were designed and cloned into the donor vector, YKO-LV002-sgBRD7 (U6/mCherry&Puro) (UbiGene, Guangzhou, China), to establish BRD7-silenced stable EBV^+^ Akata and Raji cells. The YKO-LV002-sgBRD7 vector and the YCas9-LV002 (Cas9&Hygro) vector were cotransfected with packaged plasmids into the HEK293T cells, respectively. The rest of the procedure is the same as that used for the generation of shBRD7 BL cells described above. Oligonucleotide sequences of sgBRD7 are listed in Table S1.

### Sanger sequencing.

According to the manufacturer's instructions, total genomic DNA was isolated using the Universal genomic DNA kit (CWbiotech) and quantified with a NanoDrop spectrophotometer (Thermo Fisher Scientific). PCR was carried out using Super *Pfx* DNA polymerase (CWbiotech). The conditions and protocols of PCR were based on the product information for Super *Pfx* DNA polymerase. The amplification products were analyzed by agarose gel electrophoresis and then were used for Sanger sequencing.

### Cloning and transfection.

The pCAGGS vector (71, 72) and the pCAGGS-EBNA1-Myc vector for myc-tagged EBNA1 expression were previously constructed (73). According to the manufacturer's instructions, cells were transfected with Lipofectamine 3000 (ThermoFisher Scientific). For the BiMC assay, a 1.9-kb coding sequence of BRD7 (GenBank accession no. NM_013263) was subcloned into a pCAGGS expression vector containing a C-terminal yellow fluorescent protein (YFP) tag. The rest of the procedure and the use of other vectors of pCAGGS-EBNA1-NY were followed as previously described (74).

### Coimmunoprecipitation assay.

In brief, 293T cells were transfected with EBNA1-Myc and BRD7-Flag for 48 h and lysed using RIPA lysis buffer plus PMSF for 20 min at 4°C. Cell lysates were incubated with Flag or Myc antibody at 4°C overnight. Protein A/G-conjugated magnetic beads (Bimake) were added, and the mixture was incubated for 4 h at 4°C. The immunocomplexes were washed with lysis buffer three times and subjected to immunoblotting.

### Statistical analysis.

All of the data shown were analyzed using GraphPad Prism 5 (GraphPad Software, USA), and the results were obtained from triplicate independent experiments and are presented as the mean ± standard deviation (SD). Statistical expression differences between groups were determined using the Student's *t* test or one-way analysis of variance (ANOVA). *P* values of <0.05 were deemed to be statistically significant.

### Data availability.

The BRD7 ChIP-seq data from EBV^−^ and EBV^+^ Akata cells have been deposited into the Gene Expression Omnibus (www.ncbi.nlm.nih.gov/geo/) under accession no. GSE202701.
