# Supplementary material for: Epstein-Barr Virus Synergizes with BRD7 to Conquer c-Myc-Mediated Viral Latency Maintenance via Chromatin Remodeling
Source: Microbiol Spectr. 2023 Feb 2;11(2):e01237-22. doi: 10.1128/spectrum.01237-22 (PMC10101146; doi:10.1128/spectrum.01237-22)
Supplement: Supplemental file 1 — Supplemental material. Download spectrum.01237-22-s0001.pdf, PDF file, 1.4 MB [file spectrum.01237-22-s0001.pdf]

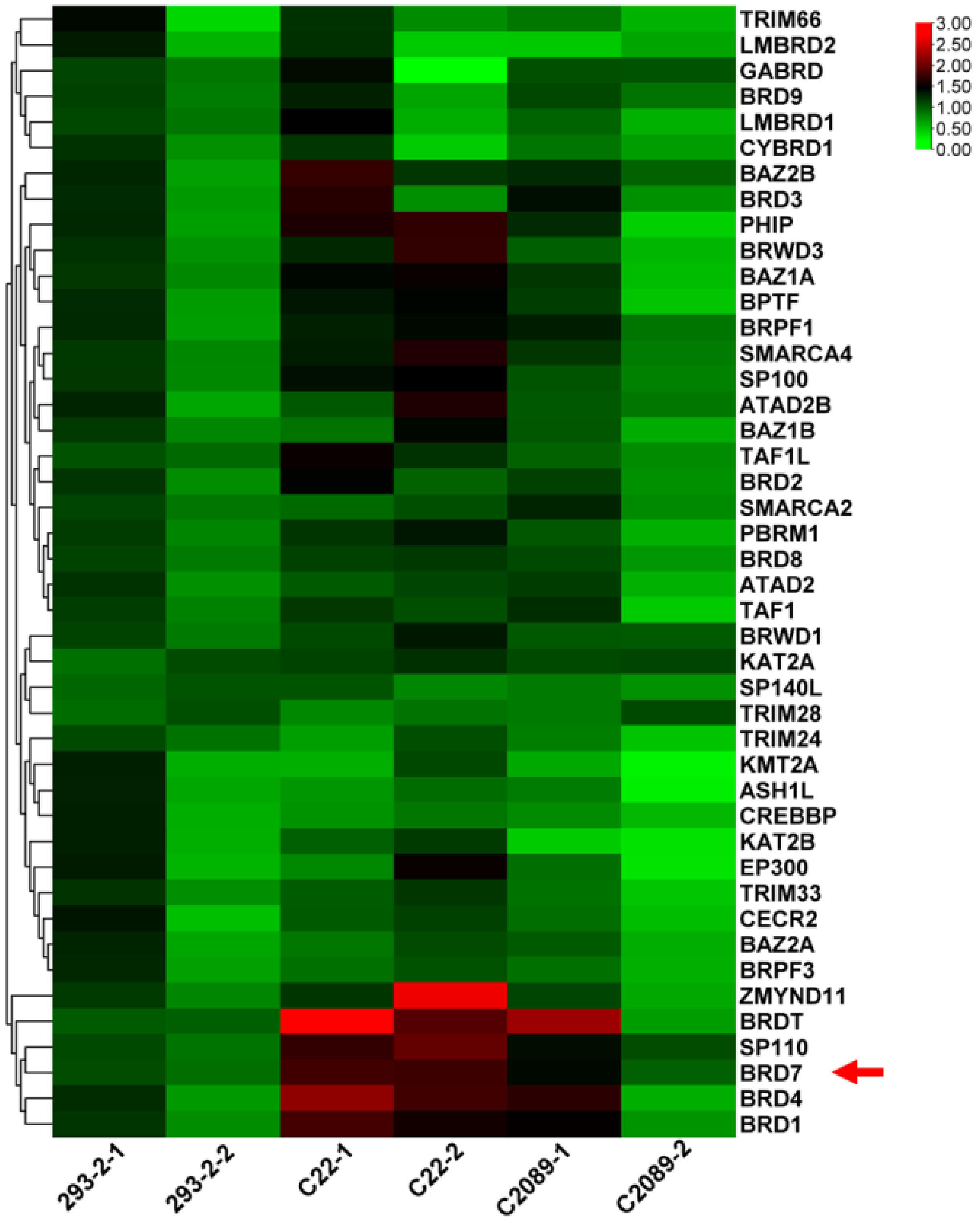

FIG S1. Heatmap depicts BRD family gene expression from EBV negative (293) and positive (293-EBV: C22 and C2089) cells. Normalize the gene expression data through log2, and use TBtools software to visualize the gene expression matrix. The gene value is low to high, green to black to red. The higher the expression, the redder the color. The red arrow indicates BRD7.

A

Mapped to human

Statistics	A- BRD7	A- Input	A+ BRD7	A+ Input
All	100265845	80194452	91984132	82340884
UnMapped	425951	80046	7384681	1148662
Mapped	99839894	80114406	84599451	81192222
MappedRate	0.996	0.999	0.920	0.986
UniqueMapped	92766198	74358173	71403798	74310948
UniqueMappedRate	0.925	0.927	0.776	0.902
MultiMapped	7073696	5756233	13195653	6881274

B

Mapped to EBV

Statistics	A+ BRD7	A+ Input
All	7327584	1106681
UnMapped	397340	82408
Mapped	6930244	1024273
MappedRate	0.946	0.926
UniqueMapped	6405248	929643
UniqueMappedRate	0.874	0.840
MultiMapped	524996	94630

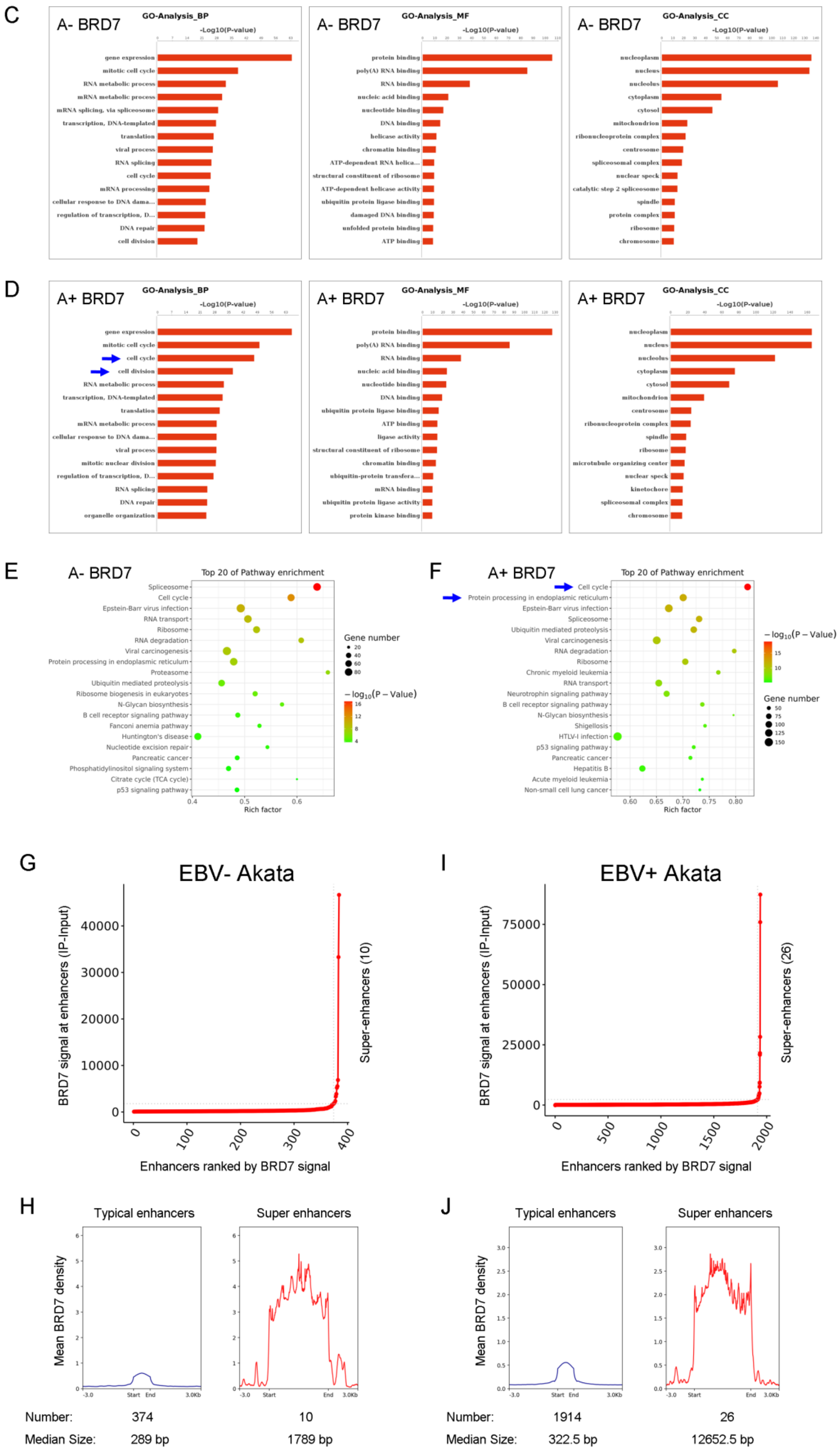

FIG S2. EBV-mediated BRD7 regulates the cellular pathways and enhancers. (A) The clean reads were aligned to human genome (hg38) sequences using the bwa program. Red lines indicate the human reads (%) in EBV+ Akata cells. (B) Total non-human reads from EBV-positive cells were aligned to Akata EBV genome (GenBank accession no. KC207813.1) sequences using the bwa program. Red lines indicate the EBV reads (%) in non-human reads of EBV+ Akata cells. All: Total clean reads involved in this alignment; Unmapped: Reads that are not aligned to the human genome; Mapped: Total human reads; Mappedrate: The ratio of human reads / all; Uniquemapped: Total unique mapped reads; Uniquemappedrate: The ratio of unique mapped reads / mapped reads; Multimapped: Total multi-mapped reads. (C and D) Top 15 gene GO analysis in biological process (BP), molecular function (MF), and cellular component (CC) of BRD7 enrichment for gene peaks near the TSSs in EBV- and EBV+ Akata cells. Blue arrows indicate the specific BRD7-enriched GO-biological processes in EBV+ Akata cells. (E and F) Top 20 pathways of BRD7 enrichment for gene peaks near TSSs in EBV- and EBV+ Akata cells. Blue arrows show the specific BRD7 enriched pathways in EBV+ Akata cells. (G-J). BRD7-related typical enhancers and super-enhancers in EBV- and EBV+ Akata cells. (G) Rank order of BRD7 ChIP-seq signal (total reads) across the 384 enhancers in EBV-negative Akata cells. (H) Metagenes of BRD7 ChIP-seq density (reads per million per base pair) across the 374 typical enhancers and the 10 super-enhancers in EBV-negative Akata cells. Metagenes were centered on the enhancer region (289 base pairs for typical enhancers and 1.7 kb for super-enhancers), with 3 kb surrounding each enhancer region. (I) Rank order of BRD7 ChIP-seq signal (total reads) across the 1940 enhancers in EBV-positive Akata cells. (J) Metagenes of BRD7 ChIP-seq density (reads per million per base pair) across the 1914 typical enhancers and the 26 super-enhancers in EBV-positive Akata cells. Metagenes were centered on the enhancer region (322.5 base pairs for typical enhancers and 12.6 kb for super-enhancers), with 3 kb surrounding each enhancer region.

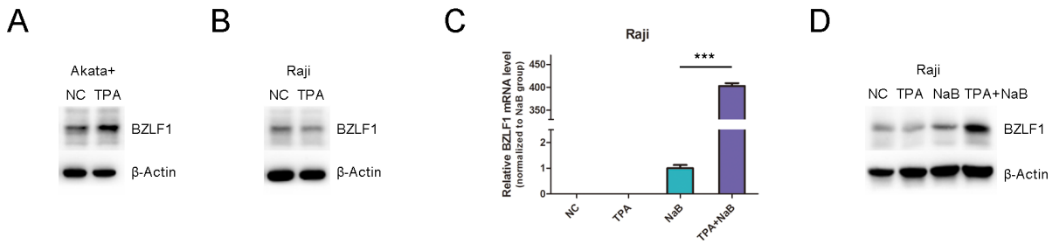

FIG S3. Detection of BZLF1 with the stimulation of EBV lytic reactivation. (A) Western blot shows the protein level of BZLF1 in EBV+ Akata cells treated with control or TPA for 24 h (hours). (B) Western blot shows the protein level of BZLF1 in Raji cells treated with control or TPA for 24 h. (C) RT-qPCR analysis of *BLZF1* mRNA level in Raji cells treated with control or the indicated reagents for 24 h. (D) Western blot shows the protein level of BZLF1 in Raji cells treated with control or the indicated reagents for 24 h.

Data are expressed as means ± SD from three independent experiments and analyzed using a two-tailed paired student t-test. ****P* < 0.001.

A

read 1	read 2
CAGCTCACCTCAGCCCGGTTCAGCCTTGT	TTAATCATTCTAGGCATCGTTTTCCTCCT
TTAGTCTAGGTCACGTTAGGTCAGTTTTG	TATGCCTCTATCATTCCTCCCTATCTACA
CCCATCTGAGTCCATTTCTGAAAGCTGGC	GCTTAGGTCAGTTTTGCCCATCTGAGTC
TGGATCATTCCTCCCTATCTACACTAACAT	CATTTCTGAAAGCTGGATGGAGTTGTCA
CCCACGCTCTGAACGCGCGCCCAATAATA	TGGCCAGAAATGGTCAGCCCACCAGAC
CCCT	CTG
150 bp	144 bp
chr.14 + chr.8	chr.8 + chr.14

B

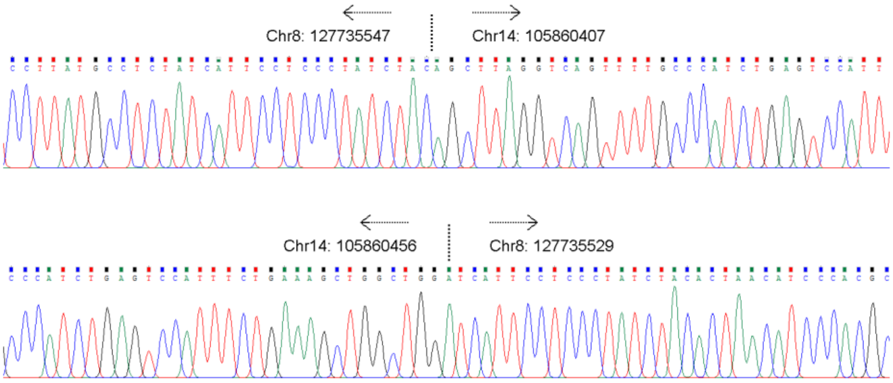

FIG S4. *c-Myc-IgH* translocation in Akata cells. (A) Two key reads are identified from BRD7 ChIP-seq data of Input groups. (B) The electropherogram depicts the junctional sequence of the *c-Myc-IgH* translocation validated using Sanger sequencing. The arrows indicate the genomic regions of each chromosomal partner and the discontinued line, the exact breakpoint of the translocation.

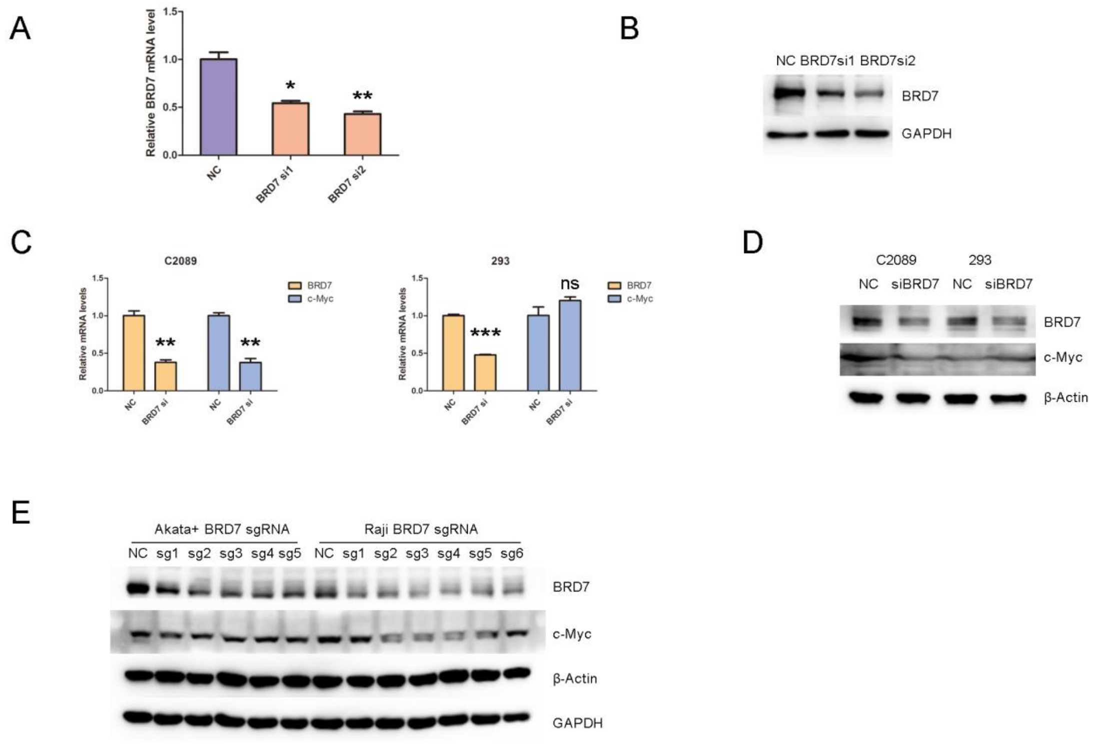

FIG S5. BRD7 regulates c-Myc expression in EBV-positive cells. (A) RT-qPCR analysis of *BRD7* mRNA level in 293 cells transfected with control or BRD7 siRNAs. (B) Western blot shows the protein level of BRD7 in 293 cells transfected with control or BRD7 siRNAs. (C) RT-qPCR analysis of *BRD7* and *c-Myc* mRNA levels in C2089 and 293 cells transfected with control or BRD7 siRNA-2. (D) Western blot shows BRD7 and c-Myc protein levels in C2089 and 293 cells transfected with control or BRD7 siRNA-2. (E) Western blot shows BRD7 and c-Myc protein levels in EBV+ Akata and Raji cells with control or sgRNA-mediated BRD7 knockout.

Data are expressed as means ± SD from three independent experiments and analyzed using a two-tailed paired student t-test. *P < 0.05; **P < 0.01; ***P < 0.001; ns, not significant.

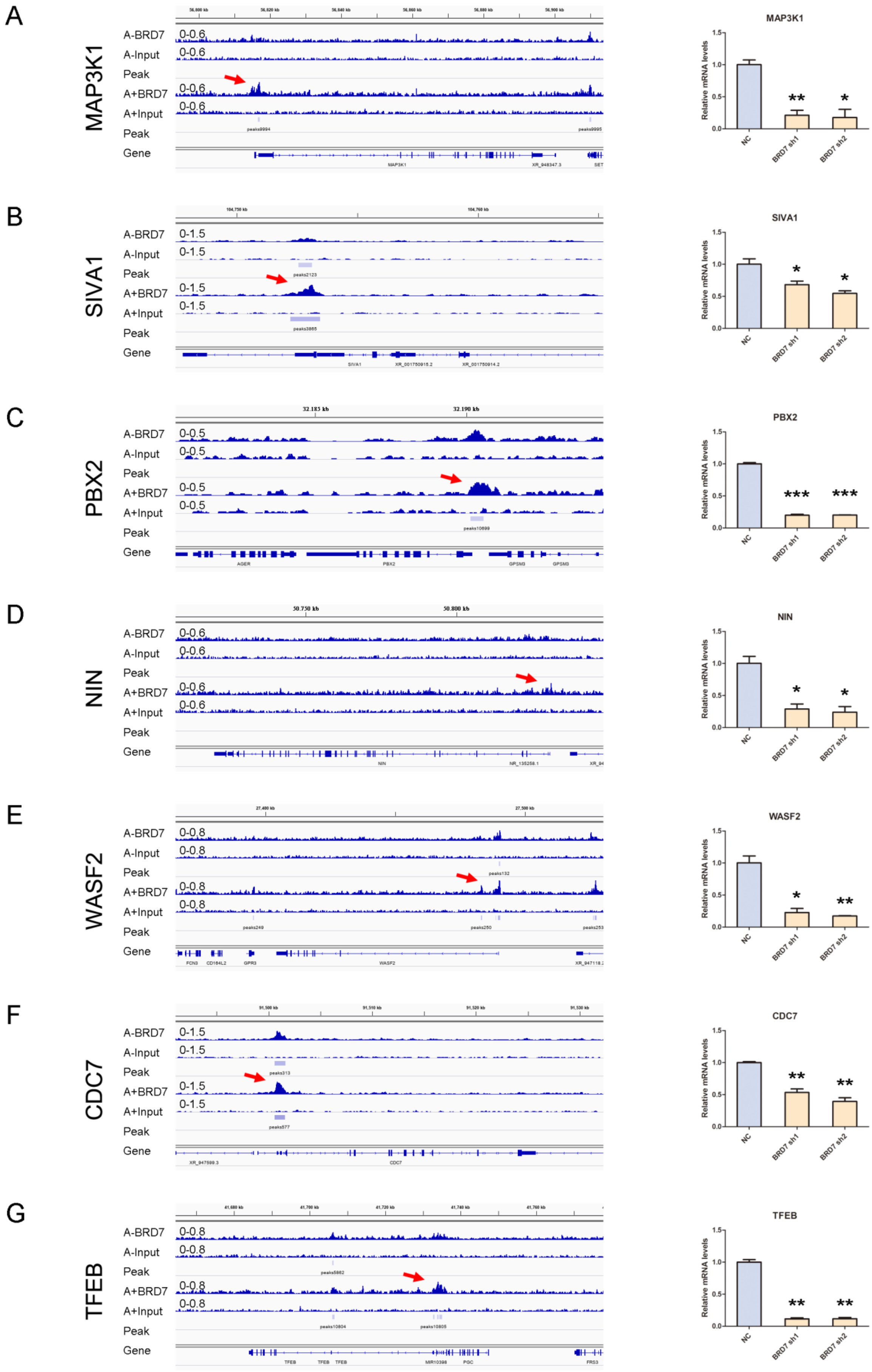
A
MAP3K1
A-BRD7
A-Input
Peak
A+BRD7
A+Input
Peak
Gene
0-0.6
0-0.6
0-0.6
0-0.6
MAP3K1
Relative mRNA levels
NC
BRD7 sh1
BRD7 sh2
**
*
B
SIVA1
A-BRD7
A-Input
Peak
A+BRD7
A+Input
Peak
Gene
0-1.5
0-1.5
0-1.5
0-1.5
SIVA1
Relative mRNA levels
NC
BRD7 sh1
BRD7 sh2
*
*
C
PBX2
A-BRD7
A-Input
Peak
A+BRD7
A+Input
Peak
Gene
0-0.5
0-0.5
0-0.5
0-0.5
32.185 kb
32.190 kb
PBX2
Relative mRNA levels
NC
BRD7 sh1
BRD7 sh2
***
***
D
NIN
A-BRD7
A-Input
Peak
A+BRD7
A+Input
Peak
Gene
0-0.6
0-0.6
0-0.6
0-0.6
50.750 kb
50.800 kb
NIN
Relative mRNA levels
NC
BRD7 sh1
BRD7 sh2
*
*
E
WASF2
A-BRD7
A-Input
Peak
A+BRD7
A+Input
Peak
Gene
0-0.8
0-0.8
0-0.8
0-0.8
WASF2
Relative mRNA levels
NC
BRD7 sh1
BRD7 sh2
*
**
F
CDC7
A-BRD7
A-Input
Peak
A+BRD7
A+Input
Peak
Gene
0-1.5
0-1.5
0-1.5
0-1.5
CDC7
Relative mRNA levels
NC
BRD7 sh1
BRD7 sh2
**
**
G
TFEB
A-BRD7
A-Input
Peak
A+BRD7
A+Input
Peak
Gene
0-0.8
0-0.8
0-0.8
0-0.8
TFEB
Relative mRNA levels
NC
BRD7 sh1
BRD7 sh2
**
**

FIG S6. BRD7 served as a cofactor of EBNA1 in the EBV latency. BRD7 binds specific sites of EBNA1-regulated genes. Tracks show BRD7 occupancy at *MAP3K1* (A), *SIVA1* (B), *PBX2* (C), *NIN* (D), *WASF2* (E), *CDC7* (F) and *TFEB* (G) in EBV+ Akata cells. Red arrows indicate the BRD7 binding sites enriched around the TSSs of genes in EBV+ Akata cells (left panel). RT-qPCR analysis of indicated mRNA levels in EBV+ Akata cells with control or BRD7 stable knockdown (right panel).

Data are expressed as means ± SD from three independent experiments and analyzed using a two-tailed paired student t-test. *P < 0.05; **P < 0.01; ***P < 0.001.

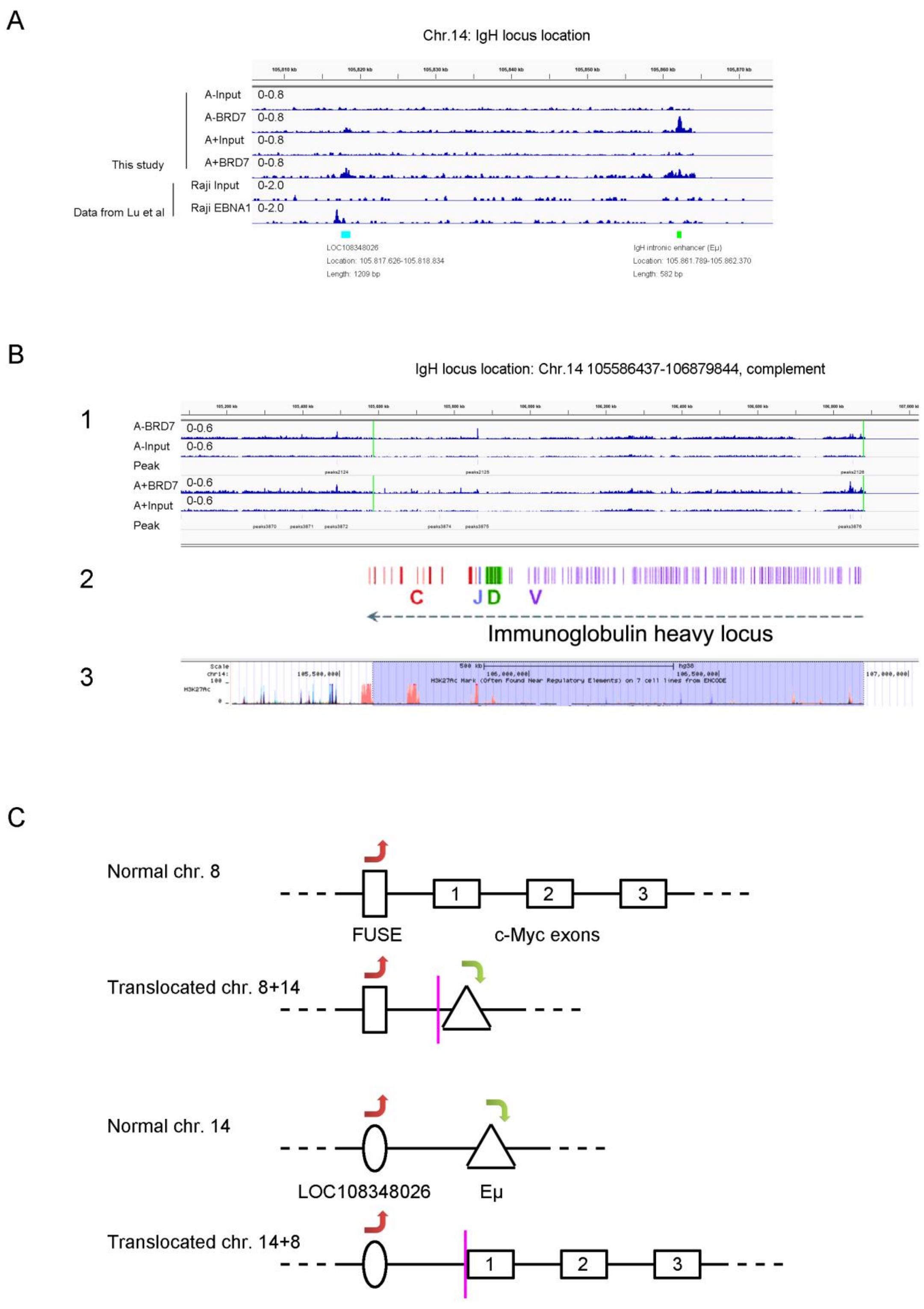

FIG S7. *c-Myc* is regulated under the control of normal and translocated locus elements. (A) Localization of BRD7 and EBNA1 ChIP-seq signals at the *IgH* locus. All ChIP-seq data were mapped to the human genome (hg38). EBNA1 ChIP-seq data are from GSM1905007. (B1) ChIP-seq for BRD7 at the *IgH* locus. Green lines show

the location of 105586437-106879844. (B2) *IgH* gene is separated into constant (C, red), joining (J, blue), diversity (D, green), and variable (V, purple) regions. (B3) H3K27ac mark on 7 cell lines from ENCODE database in the *IgH* locus. (C) Schematic diagram depicts the *c-Myc*-related regulatory elements of the normal and translocated allele in 8q24 and 14q32. Red arrows indicate BRD7 enrichments in EBV+ Akata cells compared with EBV- Akata cells. Green arrows indicate BRD7 enrichments in EBV- Akata cells compared with EBV+ Akata cells. Red lines indicate the exact breakpoint of the translocation in Akata cells.

TABLE S1. Sequence information of oligos.

TABLE S2. Sequence information of antibodies.

S1 Table. Sequences of oligos used in this study

Oligonucleotides	SOURCE	IDENTIFIER
CAGACGAGTCCGTAGAAGGGT	This paper	EBV-W-For
TAGGGAACTGAGGAGGGCAT	This paper	EBV-W-Rev
CCTTTTGTAGGAGGGACTTAGAG	This paper	gDNA-1- For
GTATTCACCACCCCACTATGC	This paper	gDNA-1-Rev
TGATGACATCAAGAAGGTGGTGAAG	This paper	gDNA-2- For
TCCTTGGAGGCCATGTGGGCCAT	This paper	gDNA-2-Rev
TCACGCCCCTGTGAGATTAGA	PrimerBank	BRD7-For
AGCCAGGAGCAATAAAATCAGTC	PrimerBank	BRD7-Rev
GGAGCGAGATCCCTCCAAAAT	PrimerBank	GAPDH-For
GGCTGTTGTCATACTTCTCATGG	PrimerBank	GAPDH-Rev
ACATCTGCTTCAACAGGAGGCG	This paper	BZLF1-For
ACAACAGCTAGCAGACATTGGTG	This paper	BZLF1-Rev
GTCAAGAGGCGAACACACAAC	PrimerBank	c-Myc-For
TTGGACGGACAGGATGTATGC	PrimerBank	c-Myc-Rev
ACCCTATGGAGAACCGAGAC	PrimerBank	CASC11-For
GAGGACCAACTCAGTAGGAAAT	PrimerBank	CASC11-Rev
CTGGCGCAGGAGTATGACAAG	PrimerBank	SMC1A-F
CCCGTACTACCTCATCCTTCA	PrimerBank	SMC1A-R
GGATAAGAAGCTAACCAAAGCCC	PrimerBank	RAD21-F
CTCCCAGTAAGAGATGTCCTGAT	PrimerBank	RAD21-R
AAAGACGCTTATTATCGGCGAG	PrimerBank	SUPT16H-F
AAAGAGCCATGTCTGTAAGGC	PrimerBank	SUPT16H-R
AAACTCACTATCGCCTTGAGC	PrimerBank	SSRP1-F
GTCACCTCATTCTTGCCTGTG	PrimerBank	SSRP1-R
CGGAGTCTTCGGATAAGCTCT	PrimerBank	PARP1-F
TTTCCATCAAACATGGGCGAC	PrimerBank	PARP1-R

AGACCAGCCAGCTAACCCTAC	PrimerBank	MED21-F
TGCGCTTTGTATCTTCTCCAG	PrimerBank	MED21-R
AGCTACAGGATAATCTTCGCCA	PrimerBank	MED30-F
TGGAATAAGTTGCTCGACTGGA	PrimerBank	MED30-R
CATCAGGTCGCACAGTGAAAT	PrimerBank	MAP3K1-F
TCAGGGCTATATGGTGAGAAGC	PrimerBank	MAP3K1-R
CAAGCGACTCCTGTTCCTCG	PrimerBank	SIVA1-F
GTCTGGTCCAATCAGCATCTG	PrimerBank	SIVA1-R
CCCTAAACTGCCACCGAATGA	PrimerBank	PBX2-F
GTGTTCGATGGAGTTGTCAGG	PrimerBank	PBX2-R
GGAAGCGTTACGGACGAAGG	PrimerBank	NIN-F
GGGAGGGGAAGATCCACTCT	PrimerBank	NIN-R
TAGTAACGAGGAACATCGAGCC	PrimerBank	WASF2-F
AAGGGAGCTTACCCGAGAGG	PrimerBank	WASF2-R
AGTGCCTAACAGTGGCTGG	PrimerBank	CDC7-F
CACGGTGAACAATACCAAACTGA	PrimerBank	CDC7-R
ACCTGTCCGAGACCTATGGG	PrimerBank	TFEB-F
CGTCCAGACGCATAATGTTGTC	PrimerBank	TFEB-R
GAAGGGGACTTGGGGTGATTTTC	This paper	ChIP-oriLyt L-F
CTTCTTGTCAACCTCTTCAGGCC	This paper	ChIP-oriLyt L-R
TGGGAGTGGTTTTGTGTGAGCC	This paper	ChIP-oriLyt R-F
GGAAAGGGAGGAAATAGAGGCC	This paper	ChIP-oriLyt R-R
GCCUCUCACAAGCUCUUUATT	This paper	siBRD7-1-sense
UAAAGAGCUUGUGAGAGGCTT	This paper	siBRD7-1- antisense
GUCCCUCAUACAGAGAAAUTT	This paper	siBRD7-2-sense
AUUUCUCUGUAUGAGGGACTT	This paper	siBRD7-2-antisense
GCCTCTCACAAGCTCTTTAGC	This paper	shBRD7-1
GGGTCCCTCATACAGAGAAAT	This paper	shBRD7-2
CCCCCTCCTCACCCTCGTAGAGG	This paper	sgBRD7

S2 Table. Antibodies used in this study

Antibodies	SOURCE	IDENTIFIER
BRD7 polyclonal antibody	Proteintech	Cat# 51009-2-AP
GAPDH Rabbit pAb (HRP Conjugate)	ZEN BIO	Cat# 380646
β-Actin (8F10) (HRP-Conjugate) Mouse mAb	ZEN BIO	Cat# 700068
BRD7 (D9K2T) Rabbit mAb (ChIP Formulated)	Cell Signaling Technology	Cat# 14910
EBV ZEBRA (BZ1)	Santa Cruz Biotechnology	Cat# sc-53904
c-Myc Rabbit pAb	ABclonal	Cat# A17332
Anti-DYKDDDDK-tag-HRP (HRP-Conjugate) Mouse mAb	ZEN BIO	Cat# 700002
Anti-Myc-Tag (9E10) Mouse mAb (HRP Conjugate)	ZEN BIO	Cat# 700003
Mouse Control IgG	ABclonal	Cat# AC011
Rabbit Control IgG	ABclonal	Cat# AC005
HRP Goat Anti-Mouse IgG (H+L)	ABclonal	Cat# AS003
HRP Goat Anti-Rabbit IgG (H+L)	ABclonal	Cat# AS014
EA-D	Millipore	Cat# MAB8186
